# Preferential Upregulation of AMOT-p80 Is Associated with YAP-Linked Resistance to 5-Fluorouracil and Oxaliplatin in Colorectal Cancer Cells

**DOI:** 10.3390/biom16060767

**Published:** 2026-05-22

**Authors:** Yeho Kim, Jin-Kyung Hong, Mina Yeom, Min-Ju Kim, Jae-Hyeon Woo, Joo-Ho Shin, Tae Hyung Won, Yunjong Lee, Jeong-Yun Choi

**Affiliations:** Department of Pharmacology, Sungkyunkwan University School of Medicine, 2066 Seobu-ro, Jangan-gu, Suwon 16419, Gyeonggi-do, Republic of Korea

**Keywords:** AMOT-p80, YAP, chemoresistance, 5-fluorouracil, oxaliplatin, colorectal cancer

## Abstract

Resistance to 5-fluorouracil (5-FU) and oxaliplatin (OXA) remains an obstacle in colorectal cancer (CRC) therapy, but the upstream mechanisms enabling adaptive survival remain unclear. Angiomotin (AMOT), a Hippo-YAP regulator, is expressed as two major isoforms, p130 and p80, but the contribution of isoform-specific AMOT regulation to chemoresistance is unknown. RNA-seq of OXA-resistant cells identified AMOT as a candidate determinant, and its isoform-specific regulation and functional relevance were then examined in OXA- and 5-FU-resistant CRC sublines. AMOT-p80 was preferentially upregulated, whereas AMOT-p130 remained largely unchanged. Common AMOT pre-mRNA was elevated, whereas p130-specific pre-mRNA was unchanged, consistent with preferential transcriptional activation favoring the p80 isoform. Functionally, AMOT depletion minimally affected basal viability but significantly sensitized resistant cells to 5-FU or OXA, with increased apoptotic responses. AMOT silencing reduced nuclear YAP and lowered c-Myc and Cyclin D1 protein levels, whereas AMOT-p80 re-expression restored nuclear YAP, with recovery of c-Myc/Cyclin D1 levels and drug tolerance. YAP knockdown attenuated these outputs and blunted the additional effect of AMOT depletion. AMOT-p80 overexpression in parental cells increased c-Myc/Cyclin D1 protein levels and enhanced tolerance to 5-FU and OXA. These findings suggest that preferential AMOT-p80 upregulation is linked to YAP-associated chemoresistant phenotypes in CRC cells.

## 1. Introduction

Chemoresistance to 5-fluorouracil (5-FU) and oxaliplatin (OXA) remains a major obstacle to effective chemotherapy for colorectal cancer (CRC). These agents disrupt nucleic acid metabolism and induce DNA damage-associated stress that can culminate in apoptosis; however, CRC cells frequently acquire multifactorial adaptations that blunt cytotoxicity, including altered drug transport and metabolism, rewired survival signaling, enhanced stress tolerance and repair capacity, and deregulated apoptosis [1,2]. Because such adaptations are rarely driven by a single pathway, identifying shared determinants that support cross-agent tolerance remains an important unmet need [1,2].

Among candidate stress-adaptive programs, the Hippo pathway effectors YAP and TAZ have emerged as central regulators of growth, plasticity, and therapy resistance across cancer types [3,4]. In many settings, increased nuclear YAP/TAZ activity sustains transcriptional programs that promote proliferation, survival, and phenotypic plasticity, including EMT-related features, thereby contributing to drug tolerance [3,5]. In CRC, elevated YAP activity has been associated with aggressive behavior and reduced therapeutic response, highlighting nuclear YAP-dependent transcription as a mechanistically and therapeutically relevant program [6,7,8]. However, the upstream mechanisms that maintain nuclear YAP signaling under chemotherapeutic stress remain incompletely defined [3].

One candidate class of upstream YAP regulators is the angiomotin (AMOT) family, which links polarity and cytoskeletal cues to Hippo–YAP signaling and can either restrain or support YAP activity depending on cellular context [9,10,11,12,13]. AMOT is expressed predominantly as two major isoforms, AMOT-p130 and AMOT-p80, which differ substantially at the N terminus [11,14]. AMOT-p130 contains an extended N-terminal region harboring multiple L/PPxY motifs that can engage YAP WW domains and influence YAP localization and transcriptional output [10,15,16]. Prior studies indicate that the effect of p130 on YAP is context-dependent, with reports supporting both junctional/cytoplasmic sequestration and attenuation of YAP activity [10,16], and, in other settings, YAP-dependent transcriptional activation and growth [15]. By contrast, AMOT-p80 lacks this N-terminal extension [11,14] and has been associated with cytoskeletal remodeling, motility-related phenotypes, and, in some contexts, enhanced nuclear YAP activity and YAP-responsive gene induction [14,17]. Together, these observations support functional divergence between AMOT isoforms and raise the possibility that altered AMOT isoform regulation may modulate nuclear YAP-dependent stress-adaptive programs.

Importantly, altered AMOT isoform output need not simply reflect a uniform increase in total AMOT expression. Because AMOT-p130 and AMOT-p80 arise from distinct transcript architectures, their relative abundance may be shaped by isoform-biased transcriptional or post-transcriptional regulation rather than by classical isoform switching alone. Thus, in the context of acquired chemoresistance, it remains unresolved whether AMOT is globally induced, whether this induction preferentially favors one isoform, and how such regulation relates to nuclear YAP-associated outputs in CRC cells.

To identify determinants of acquired OXA resistance, we performed RNA-seq profiling of parental SNUC5 CRC cells and the OXA-resistant subline SNUC5/OXTR. This unbiased screen highlighted AMOT as a highly upregulated candidate, prompting isoform-resolved investigation. We subsequently examined AMOT regulation in both SNUC5/OXTR and the independently established 5-FU-resistant subline SNUC5/5FUR, thereby assessing whether the identified AMOT pattern was shared across two distinct chemoresistant CRC models, using isoform-specific qRT-PCR and immunoblotting together with analyses of common and isoform-directed pre-mRNA species. Here, we found that chemoresistant CRC cells exhibit transcriptional activation of AMOT coupled to preferential upregulation of the p80 isoform, whereas p130 expression remains largely unchanged. We further observed that AMOT-p80 upregulation is associated with 5-FU and OXA resistance and nuclear YAP-linked transcriptional outputs. These findings identify a transcriptionally driven, p80-biased mode of AMOT regulation in chemoresistant CRC cells and provide a basis for further investigation of AMOT-p80 as a candidate marker and putative adaptive mechanism in chemotherapy resistance.

## 2. Materials and Methods

### 2.1. Cell Culture

Human colorectal cancer SNUC5 cells were obtained from the Korean Cell Line Bank (Seoul, Republic of Korea). Two chemoresistant derivative sublines derived from this parental background, the acquired 5-fluorouracil (5-FU)-resistant (SNUC5/5FUR) and oxaliplatin (OXA)-resistant (SNUC5/OXTR) cells, were kindly provided by the Research Center for Resistant Cells, Chosun University (Gwangju, Republic of Korea), where they had been established by long-term drug selection as previously described [18]. Cells were cultured in RPMI-1640 (Gibco, Grand Island, NY, USA) supplemented with 10% fetal bovine serum (FBS; Gibco) and 1% penicillin–streptomycin (Gibco) at 37 °C in a humidified incubator with 5% CO_2_. To maintain the resistant phenotype, SNUC5/5FUR and SNUC5/OXTR cells were routinely cultured in medium containing 140 μM 5-FU or 7 μM OXA (both from Sigma, St. Louis, MO, USA), respectively, twice weekly. Prior to all experiments, resistant sublines were cultured in drug-free medium for at least 48 h to minimize drug carryover.

### 2.2. RNA Sequencing and Differential Gene Expression Analysis

Total RNA (1 μg per sample; *n* = 3 biological replicates per group) was isolated from parental SNUC5 and SNUC5/OXTR cells. RNA-seq libraries were prepared using the SMARTer Ultra-Low RNA Kit (Clontech, Mountain View, CA, USA). Single-end sequencing (50 bp) was performed on an Illumina HiSeq 2000 platform (Illumina, San Diego, CA, USA), and initial raw sequence quality control was performed by Macrogen Inc. (Seoul, Republic of Korea). Reads were aligned to the human reference genome (GRCh38; Ensembl release 98) using HISAT2 v2.1.0, and transcript abundance was estimated using StringTie v2.1.3b for differential gene expression analysis between SNUC5 and SNUC5/OXTR cells. *p*-values were adjusted for multiple testing using the Benjamini–Hochberg false discovery rate method. Differentially expressed genes were defined as those with an absolute log2 fold change ≥ 1 and an adjusted *p* value < 0.05. For visualization, volcano plots were generated by plotting the log2 fold change on the x-axis and the −log10 adjusted *p* value on the y-axis.

### 2.3. Quantitative Reverse Transcription PCR (qRT-PCR)

Total RNA was isolated using the TRIzol reagent (Invitrogen, Carlsbad, CA, USA), and cDNA was synthesized with the iScript cDNA Synthesis Kit (Bio-Rad, Hercules, CA, USA) according to the manufacturers’ instructions. qRT-PCR was performed on a Bio-Rad iQ5 Real-Time PCR Detection System using iQ SYBR Green Master Mix (Bio-Rad). Thermal cycling conditions were 95 °C for 10 min, followed by 40 cycles of 95 °C for 15 s and 60 °C for 1 min. For mature transcript analysis, primer sets were used to quantify common AMOT, AMOT-p80, and AMOT-p130 transcripts. Primers for AMOT-p80 and AMOT-p130 were designed to detect isoform-specific exon–exon junctions, whereas the common AMOT assay measured a shared mature transcript region. Primer sequences were as follows: AMOT-p80 (F 5′-TCCAATGCTGGATCAGGCTTGC-3′, R 5′-ATTCAGCACGGTAGTCTCCACC-3′), AMOT-p130 (F 5′-GCCAGTGATGAAGAGGACAA-3′, R 5′-CTTGTCTGTGGTCTTGGTGC-3′), AMOT (common) (F 5′-AAGCGTTGCCTTGACATGGAG-3′, R 5′-GGAACGCTGCTGGAGTACTTTGA-3′), and ACTB (F 5′-CACCTTCTACAATGAGCTGCGTGT-3′, R 5′-CACAGCCTGGATAGCAACGTACA-3′). For precursor transcript analysis, two additional primer sets were used: a common AMOT pre-mRNA assay designed to detect a shared precursor region (F 5′-GGACCAAAGACGACACATCG-3′, R 5′-TGCTACCTGAGGAAACCATACC-3′) and an AMOT-p130 pre-mRNA assay designed to selectively detect the p130-directed precursor transcript (F 5′-GCCAGGGCACTTCTATAGTGAG-3′, R 5′-TCTTACCTGGCTGCTCCATAC-3′). Relative expression levels were calculated using the 2^−ΔΔCt^ method with ACTB as the internal control. Each sample was analyzed in triplicate, and data are presented as mean ± SD.

### 2.4. Western Blotting

Total cell lysates were prepared in RIPA buffer containing protease and phosphatase inhibitors. For nuclear fractionation, nuclear and cytoplasmic extracts were prepared using NE-PER™ Nuclear and Cytoplasmic Extraction Reagents (Thermo Fisher Scientific, Waltham, MA, USA; Cat. No. 78833) according to the manufacturer’s protocol. Equal amounts of protein (30 μg) were separated by 7.5–8% SDS-PAGE and transferred to PVDF membranes (Millipore, Billerica, MA, USA). Membranes were blocked with 5% non-fat milk and incubated with primary antibodies against AMOT (Cell Signaling Technology, Danvers, MA, USA; Cat. No. 43130), YAP (Cell Signaling Technology; Cat. No. 12395), c-Myc (Santa Cruz Biotechnology, Dallas, TX, USA; Cat. No. sc-47694), Cyclin D1 (Santa Cruz Biotechnology; Cat. No. sc-8396), Slug/SNAI2 (Santa Cruz Biotechnology; Cat. No. sc-166476), ZEB1 (Santa Cruz Biotechnology; Cat. No. sc-25388), and TWIST1 (Santa Cruz Biotechnology; Cat. No. sc-15393). β-Actin (Abcam, Cambridge, UK; Cat. No. ab8227) and TBP (Abcam; Cat. No. ab28175) were used as loading controls for whole-cell and cytoplasmic fractions and nuclear fractions, respectively. Signals were visualized using HRP-conjugated secondary antibodies (goat anti-rabbit IgG [H+L]-HRP, Thermo Fisher Scientific/Invitrogen; Cat. No. 31460; and goat anti-mouse IgG [H+L]-HRP, Thermo Fisher Scientific/Invitrogen; Cat. No. 31430), followed by ECL detection using an ECL reagent (GenDEPOT, Katy, TX, USA) and imaging on a ChemiDoc system (Bio-Rad). AMOT-p80 was detected using Cat. No. 43130. Because AMOT-p130 was not consistently detectable under our experimental conditions, AMOT-p130 was evaluated on a separate blot using a full-length AMOT-preferential antibody (Cell Signaling Technology; Cat. No. 51142). Band intensities were quantified using ImageJ (version 1.54; NIH, Bethesda, MD, USA) and normalized to β-actin or TBP, as indicated. For many immunoblots, target proteins and the corresponding loading controls were assessed on parallel membranes prepared from the same lysates, loaded with equal protein amounts, electrophoresed and transferred in parallel, and processed under matched conditions [19]. Therefore, the corresponding densitometric analyses were interpreted as semi-quantitative estimates of relative protein abundance, rather than absolute quantitative measurements, and were used to support qualitative assessment of the immunoblot patterns. Values were expressed as fold change relative to the indicated control (set to 1.0).

### 2.5. siRNA Transfection

Cells were transfected with 20 nM control siRNA (siControl) or AMOT-targeting siRNAs (Bioneer, Daejeon, Republic of Korea): siAMOT#1 (siRNA No. 154796-1; targeting the AMOT coding sequence [CDS]) and siAMOT#2 (siRNA No. 154796-2; targeting the 3′ untranslated region [3′UTR]). For YAP knockdown, a YAP1-targeting siRNA (siYAP; AccuTarget™ Genome-wide Predesigned siRNA, BioRP grade; Bioneer) was used at 20 nM. Transfections (single siRNA or the indicated combinations) were performed using Lipofectamine RNAiMAX (Invitrogen, Thermo Fisher Scientific) according to the manufacturer’s instructions. The 20 nM concentration was used to achieve consistent target knockdown and reproducible phenotypic effects in SNUC5-derived cells while maintaining acceptable cell viability. Both siAMOT#1 and siAMOT#2 were designed to reduce total AMOT expression. Knockdown efficiency was assessed at 48 h post-transfection by isoform-specific RT-qPCR and/or Western blotting.

### 2.6. AMOT-p80 Overexpression and Rescue Experiments

An AMOT-p80 expression plasmid (pRP[Exp]-Neo-CMV>hAMOT [NM_133265.5]/3xGGGGS/FLAG; Vector ID: VB250922-1538uca; VectorBuilder Inc., Chicago, IL, USA) and the corresponding empty vector (EV) control were used for ectopic expression in parental SNUC5 cells. For rescue experiments, resistant sublines were co-transfected with siAMOT#2 (3′UTR-targeting) and the AMOT-p80 expression plasmid lacking the targeted 3′UTR, thereby enabling siRNA-resistant re-expression of AMOT-p80. Plasmid transfection was performed using Lipofectamine 3000 (Thermo Fisher Scientific, Waltham, MA, USA), according to the manufacturer’s instructions. AMOT-p80 overexpression and re-expression were confirmed by Western blotting.

### 2.7. Cell Viability Assay (MTT)

Cell viability was assessed using an MTT assay. Cells were seeded in 96-well plates (5 × 10^3^ cells/well) and allowed to adhere overnight. At 24 h post-transfection, cells were treated with 5-FU (9 μM) or OXA (3 μM) for 48 h. These concentrations were selected to approximate the IC_50_ in parental SNUC5 cells based on preliminary dose–response experiments and to provide measurable but non-saturating cytotoxic stress that allowed comparison of drug responses between parental and resistant cells. Following drug treatment, MTT (0.5 mg/mL; Sigma) was added for 4 h, and formazan crystals were dissolved in DMSO (Sigma). Absorbance was measured at 570 nm, and viability was expressed as a percentage relative to the untreated control.

### 2.8. Apoptosis Assays

Apoptosis was evaluated by nuclear morphology, Sub-G1 analysis, and caspase-3 activity. For nuclear staining, cells were incubated with Hoechst 33342 (10 μg/mL; Thermo Fisher Scientific) for 10 min at 37 °C, and apoptotic nuclei were identified based on chromatin condensation and nuclear fragmentation. For Sub-G1 quantification, cells were fixed in 70% ethanol, stained with propidium iodide (PI; 100 μg/mL, Sigma) in the presence of RNase A (100 μg/mL; Thermo Fisher Scientific) and EDTA (2 mM; Bioneer) in PBS (Welgene, Gyeongsan, Republic of Korea), and analyzed by flow cytometry (BD Biosciences, San Jose, CA, USA). Caspase-3 activity was measured using a colorimetric assay kit (Abcam; Cat. No. ab39401) according to the manufacturer’s instructions. Data are presented as mean ± SD from three independent experiments.

### 2.9. Protein–Protein Interaction Assays (Co-Immunoprecipitation and PLA)

For co-immunoprecipitation (co-IP), whole-cell lysates were incubated with antibodies against AMOT or YAP (or IgG control) overnight at 4 °C, followed by capture with Protein A/G PLUS agarose beads (Santa Cruz Biotechnology). Immunoprecipitates were washed and analyzed by Western blotting to detect AMOT and YAP and assess AMOT–YAP association. For proximity ligation assays (PLA), cells were fixed with 4% paraformaldehyde (Sigma), permeabilized with 0.1% Triton X-100 (Sigma), and incubated with rabbit anti-AMOT (Cell Signaling Technology; Cat. No. 43130) and mouse anti-YAP (Cell Signaling Technology; Cat. No. 12395) for 2 h at room temperature. After washing, Duolink PLA probes (Sigma) were applied for 1 h at 37 °C, followed by ligation (30 min) and amplification (100 min at 37 °C) according to the manufacturer’s instructions. Nuclei were counterstained with DAPI (Sigma) and mounted. PLA signals were imaged using a Zeiss LSM 510 confocal microscope (Carl Zeiss, Jena, Germany) and quantified as puncta per cell (≥50 cells per condition) from three independent experiments.

### 2.10. Cell Migration Assay (Scratch-Wound)

Cell migration was assessed using a scratch-wound assay. Cells were seeded in 6-well plates and cultured to near confluence. A linear wound was created using a sterile pipette tip, and detached cells were removed by washing with PBS. To minimize the contribution of cell proliferation, cells were treated with mitomycin C (10 μg/mL; Sigma) for 2 h and then maintained in low-serum medium (2% FBS). Images were acquired at 0 h and 48 h using a phase-contrast microscope (Olympus, Tokyo, Japan). Wound closure was quantified using ImageJ by calculating the remaining wound area as (wound area at 48 h/wound area at 0 h) × 100.

### 2.11. Statistical Analysis

All experiments were performed independently at least three times. Data are presented as mean ± SD. Statistical analyses were performed using GraphPad Prism 10.0 (GraphPad Software, San Diego, CA, USA). Differences among groups were assessed by one-way or two-way analysis of variance (ANOVA) with Tukey’s post hoc test, as appropriate. A *p* value < 0.05 was considered statistically significant. Statistical significance is indicated by asterisks in the figures (* *p* < 0.05).

## 3. Results

### 3.1. Drug-Resistant Cells Exhibit Transcriptionally Activated, p80-Biased AMOT Expression

**Figure 1:**
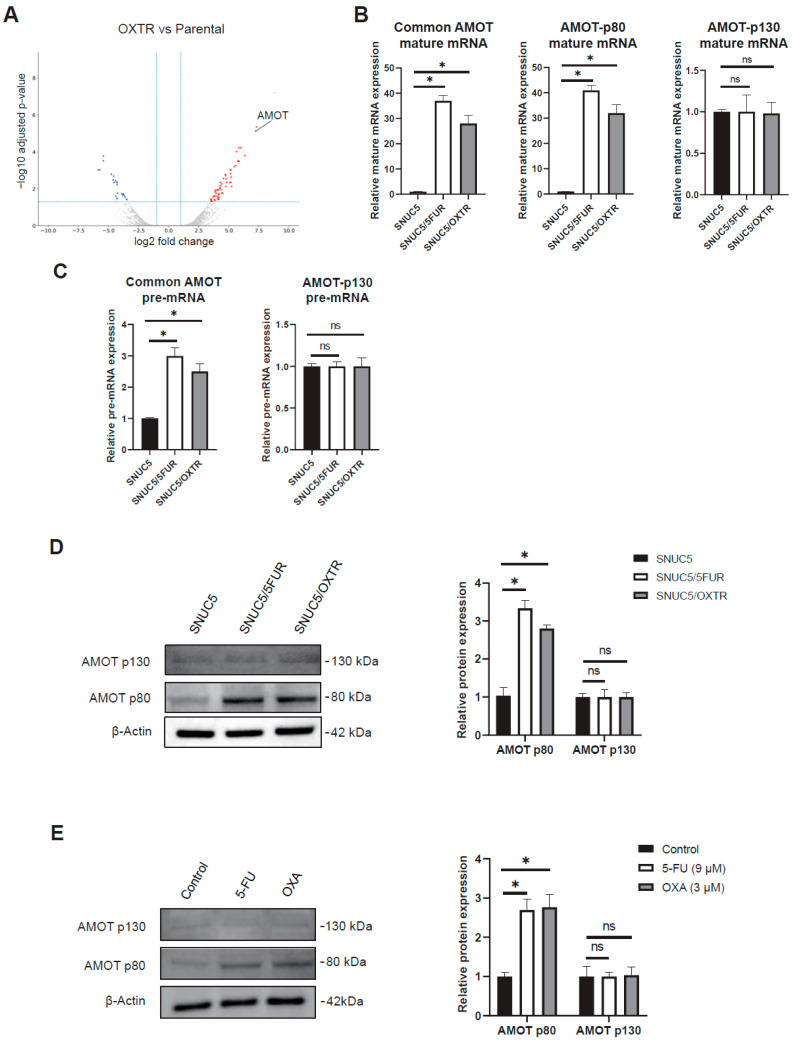
Transcriptional activation of AMOT is associated with preferential upregulation of AMOT-p80 in 5-FU- and oxaliplatin-resistant SNUC5 sublines. (**A**) Volcano plot of differential gene expression in SNUC5/OXTR versus parental SNUC5 cells from RNA-seq analysis (x-axis, log2 fold change; y-axis, −log10 adjusted *p* value), highlighting AMOT as an upregulated candidate. Red and blue dots indicate significantly upregulated and downregulated genes, respectively. (**B**) qRT-PCR analysis of common AMOT, AMOT-p80, and AMOT-p130 mature transcripts in SNUC5, SNUC5/5FUR, and SNUC5/OXTR cells, normalized to ACTB. (**C**) qRT-PCR analysis of common AMOT pre-mRNA and AMOT-p130-specific pre-mRNA in SNUC5, SNUC5/5FUR, and SNUC5/OXTR cells, normalized to ACTB. (**D**) Immunoblot analysis of AMOT isoforms in SNUC5, SNUC5/5FUR, and SNUC5/OXTR cells, with corresponding semi-quantitative densitometric estimates normalized to β-actin. (**E**) Immunoblot analysis of AMOT isoforms in parental SNUC5 cells following 48 h treatment with 5-FU (9 μM) or oxaliplatin (OXA; 3 μM), with corresponding semi-quantitative densitometric estimates normalized to β-actin. * *p* < 0.05; ns, not significant.

To identify candidate determinants of acquired oxaliplatin resistance, we performed RNA sequencing (RNA-seq) comparing parental SNUC5 cells with the oxaliplatin-resistant subline SNUC5/OXTR. A volcano plot identified AMOT as a highly upregulated candidate (Figure 1A), prompting isoform-resolved validation in both SNUC5/OXTR and the independently established 5-FU-resistant subline SNUC5/5FUR to determine whether this pattern was shared across two chemoresistant SNUC5-derived sublines. qRT-PCR analysis showed that common AMOT mRNA was significantly increased in both resistant sublines (Figure 1B). Isoform-specific analysis further revealed that this increase was not evenly distributed across AMOT isoforms: AMOT-p80 mRNA was markedly elevated, whereas AMOT-p130 mRNA remained largely unchanged (Figure 1B). To further examine the basis of this isoform bias, we measured common AMOT pre-mRNA and AMOT p130-specific pre-mRNA. Common AMOT pre-mRNA was increased in both resistant sublines, whereas p130-specific pre-mRNA was not significantly altered (Figure 1C), consistent with preferential transcriptional activation favoring the p80 isoform rather than a uniform increase across AMOT transcripts. Consistent with the transcript data, immunoblotting showed a similar pattern at the protein level, with increased AMOT-p80 and largely unchanged AMOT-p130 in both resistant sublines (Figure 1D and Figure S1). To determine whether this pattern could also be induced by acute drug exposure, parental SNUC5 cells were treated with 5-FU or oxaliplatin (OXA) at near-IC_50_ concentrations. Under these conditions, immunoblotting showed increased AMOT-p80 protein, whereas AMOT-p130 remained largely unchanged (Figure 1E and Figure S2), suggesting that p80-dominant AMOT regulation can also emerge under acute chemotherapeutic stress. Together, these results indicate that resistance to either 5-FU or OXA is associated with transcriptionally activated AMOT expression coupled to preferential upregulation of the p80 isoform, thereby motivating functional analysis of AMOT in the drug-resistant phenotype.

### 3.2. AMOT Knockdown Restores Drug Sensitivity with Increased Apoptotic Responses

**Figure 2:**
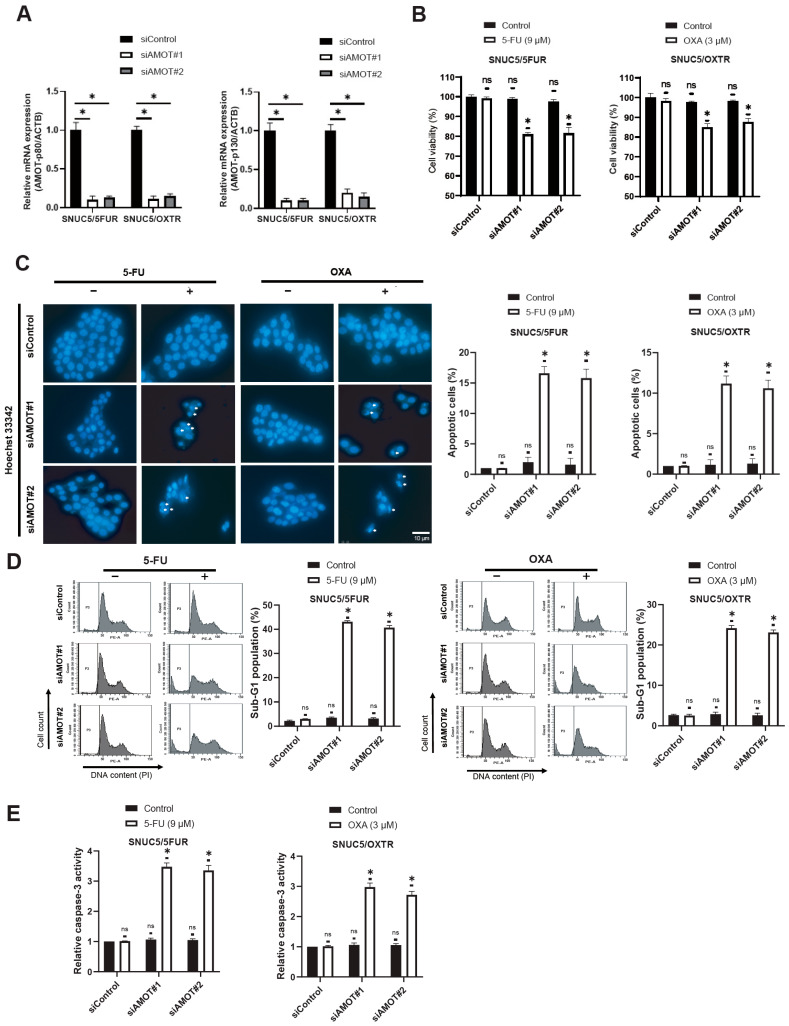
AMOT depletion sensitizes resistant cells to 5-FU or oxaliplatin and increases drug-induced apoptotic responses. SNUC5/5FUR and SNUC5/OXTR cells were transfected with siControl or siAMOT#1/#2 and treated with 5-FU (9 μM) or oxaliplatin (OXA; 3 μM) for 48 h. (**A**) Isoform-specific qRT-PCR analysis confirming knockdown of AMOT-p80 and AMOT-p130 mRNA. (**B**) Cell viability assessed by MTT assay. (**C**) Hoechst 33342 staining showing apoptotic nuclei (arrows) and quantification of apoptotic nuclei. (**D**) Sub-G1 fraction quantified by PI staining and flow cytometry. (**E**) Caspase-3 activity. * *p* < 0.05; ns, not significant.

To test whether AMOT contributes to the chemoresistant phenotype, we silenced AMOT in resistant cells using two independent siRNAs (siAMOT#1 and siAMOT#2) designed to reduce total AMOT expression. Isoform-specific qRT-PCR confirmed efficient depletion of both AMOT-p80 and AMOT-p130 transcripts in SNUC5/5FUR and SNUC5/OXTR cells (Figure 2A). AMOT silencing alone did not significantly affect basal viability in either resistant subline (Figure 2B). In contrast, upon treatment with 5-FU (9 μM) or OXA (3 μM), AMOT-depleted cells showed a significant reduction in viability relative to siControl-expressing cells exposed to the same drugs (Figure 2B). These findings indicate that AMOT depletion increases chemotherapy-induced cytotoxicity rather than causing overt basal toxicity. Consistent with this sensitization, apoptotic phenotypes, including chromatin condensation (Hoechst 33342 staining), increased Sub-G1 population, and elevated caspase-3 activity, were observed predominantly under combined AMOT knockdown and drug exposure, but not with AMOT knockdown alone (Figure 2C–E). Together, these data indicate that AMOT depletion sensitizes resistant cells to 5-FU or OXA with increased apoptotic responses. We next examined whether this AMOT-dependent phenotype involves YAP signaling.

### 3.3. The p80-Dominant AMOT State Associates with YAP and YAP-Linked Proliferative Outputs in Resistant Cells

**Figure 3:**
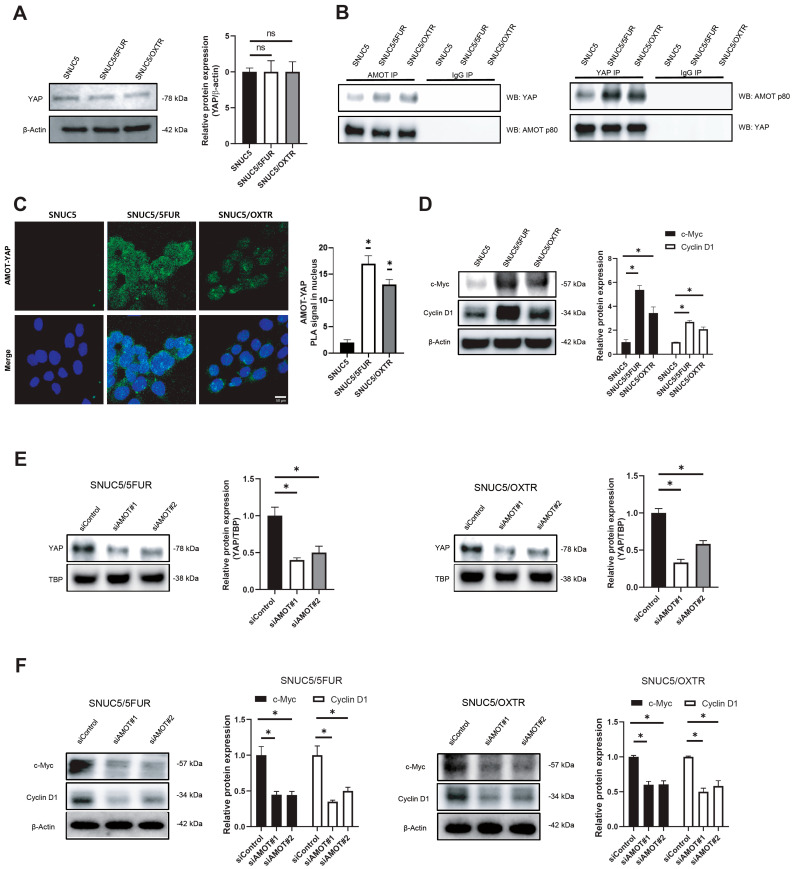
AMOT–YAP association and YAP-linked proliferative outputs in resistant cells. (**A**) Immunoblot analysis of total YAP protein expression in SNUC5, SNUC5/5FUR, and SNUC5/OXTR cells, with semi-quantitative densitometric estimates normalized to β-actin. (**B**) AMOT–YAP association assessed by co-immunoprecipitation (AMOT IP or YAP IP) followed by immunoblotting for AMOT-p80 and YAP. (**C**) Proximity ligation assay (PLA) showing nuclear AMOT–YAP proximity signals and quantification of nuclear PLA puncta. Green puncta indicate AMOT–YAP PLA signals, and blue staining indicates DAPI-stained nuclei. (**D**) Immunoblot analysis of c-Myc and Cyclin D1 protein expression in parental and resistant cells, with semi-quantitative densitometric estimates normalized to β-actin. (**E**,**F**) Effects of siAMOT#1/#2 in resistant cells on nuclear YAP (**E**); normalized to TBP) and c-Myc/Cyclin D1 (**F**); semi-quantitative densitometric estimates normalized to β-actin). * *p* < 0.05; ns, not significant.

Because AMOT knockdown enhanced drug-induced cytotoxicity and apoptosis in resistant cells, we examined whether AMOT-p80 upregulation is associated with altered Hippo–YAP signaling. Immunoblotting showed that total YAP abundance was generally comparable between parental and resistant cells (Figure 3A and Figure S3), suggesting that differences in YAP output may reflect changes in protein association and/or subcellular localization rather than overall YAP expression. Co-immunoprecipitation demonstrated AMOT–YAP association in resistant cells (Figure 3B, Figures S4 and S5), and proximity ligation assays (PLA) revealed increased AMOT–YAP proximity, with signals enriched in the nuclear compartment of resistant cells (Figure 3C). Consistent with increased YAP-linked proliferative output, c-Myc and Cyclin D1 protein levels were higher in resistant sublines than in parental cells (Figure 3D and Figure S6). Conversely, AMOT knockdown reduced nuclear YAP (YAP/TBP) (Figure 3E, Figures S7 and S8) and lowered c-Myc and Cyclin D1 protein levels (Figure 3F, Figures S9 and S10). Together, these data support the interpretation that, in the p80-upregulated resistant context, AMOT is associated with nuclear YAP abundance and YAP-linked proliferative outputs. We next examined whether AMOT–YAP-linked signaling was also associated with EMT-related transcriptional programs and motility in resistant cells.

### 3.4. AMOT Is Associated with EMT-Related Transcription Factor Expression and Motility in Resistant Cells

**Figure 4:**
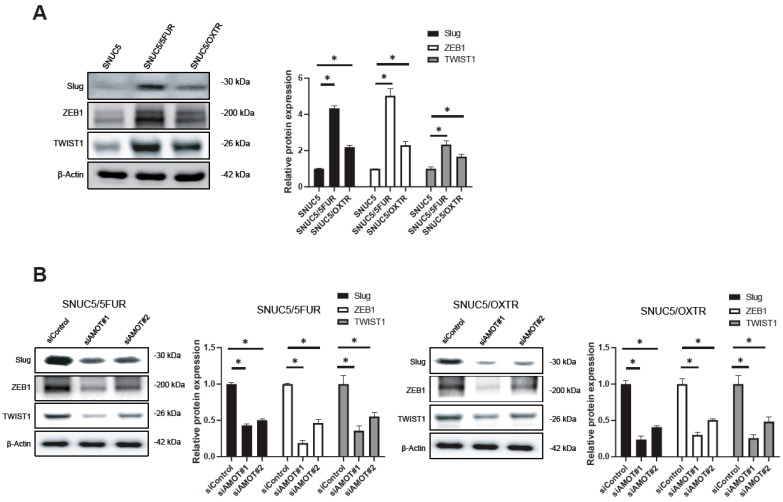

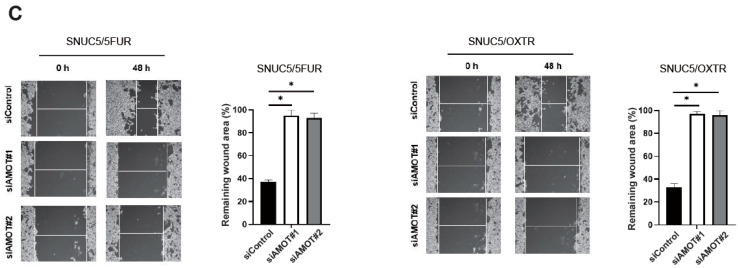
AMOT is associated with EMT-related transcription factors and motility in resistant cells. (**A**) Immunoblot analysis of SLUG, ZEB1, and TWIST1 protein expression in SNUC5, SNUC5/5FUR, and SNUC5/OXTR cells, with semi-quantitative densitometric estimates normalized to β-actin. (**B**) Immunoblot analysis of SLUG, ZEB1, and TWIST1 protein expression after transfection with siAMOT#1 or siAMOT#2 in resistant cells, with semi-quantitative densitometric estimates normalized to β-actin. (**C**) Scratch-wound assay performed under low-serum conditions (2% FBS) after mitomycin C pretreatment (10 μg/mL, 2 h). Remaining wound area (%) was calculated as (wound area at 48 h/wound area at 0 h) × 100. * *p* < 0.05.

Given the reported links between YAP signaling, EMT-associated programs, and motility [20,21], we examined whether the chemoresistant state characterized by AMOT-p80 upregulation (Figure 1) is accompanied by EMT-related transcriptional changes. SLUG, ZEB1, and TWIST1 protein levels were higher in both 5-FU- and OXA-resistant sublines than in parental SNUC5 cells (Figure 4A and Figure S11). Conversely, AMOT knockdown with either siAMOT#1 or siAMOT#2 resulted in reduced levels of these transcription factors in both resistant lines (Figure 4B, Figures S12 and S13). We next assessed whether these molecular changes were linked to altered motility using a scratch-wound assay performed under low-serum conditions with mitomycin C pretreatment. AMOT-depleted cells displayed impaired wound closure, reflected by a greater remaining wound area at 48 h relative to siControl cells (Figure 4C). Collectively, these findings indicate that, in the p80-upregulated resistant context, AMOT is associated with EMT-related transcription factor expression and motility.

### 3.5. AMOT-p80 Re-Expression Rescues YAP-Linked Outputs and Drug Tolerance in Resistant Cells

**Figure 5:**
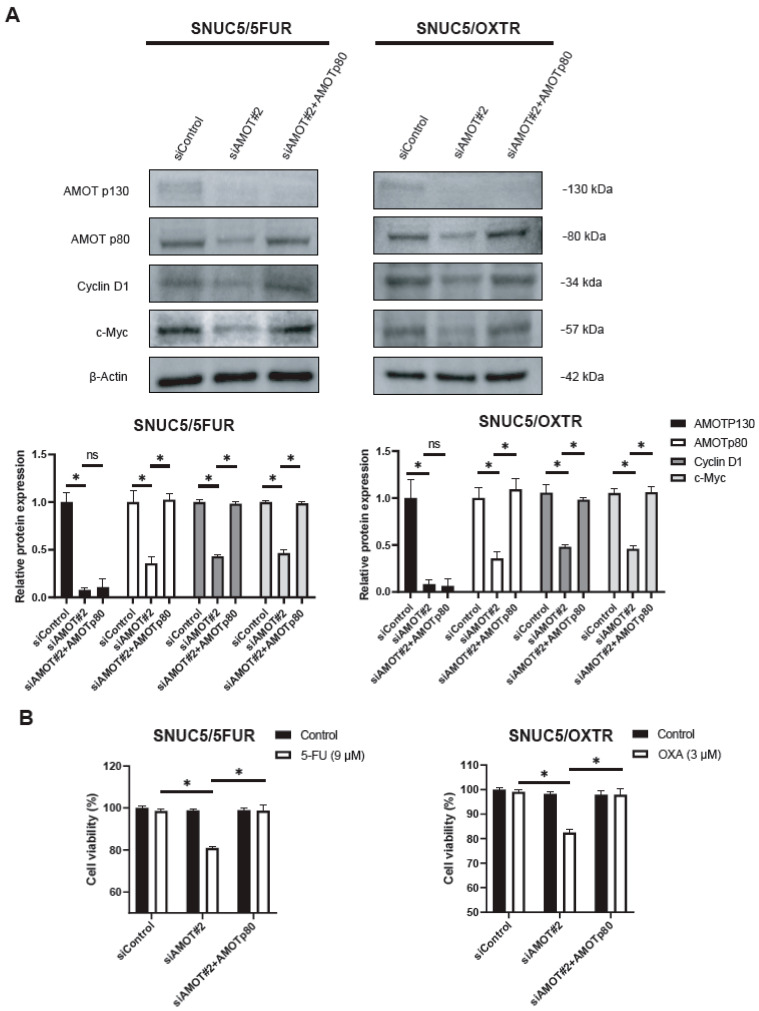
AMOT-p80 re-expression rescues YAP-linked outputs and drug tolerance in resistant cells. (**A**) SNUC5/5FUR and SNUC5/OXTR cells were transfected with siControl, siAMOT#2 (3′UTR-targeting), or siAMOT#2 plus an siRNA-resistant AMOT-p80 expression plasmid. AMOT-p80, AMOT-p130, Cyclin D1, and c-Myc protein levels were assessed by immunoblotting, with semi-quantitative densitometric estimates normalized to β-actin. (**B**) Cell viability (MTT) after 48 h treatment with 5-FU (9 μM) in SNUC5/5FUR cells (**left**) or oxaliplatin (OXA; 3 μM) in SNUC5/OXTR cells (**right**) under the same transfection conditions. * *p* < 0.05; ns, not significant.

To further test whether the observed phenotypes were linked to AMOT depletion and to assess the contribution of the p80 isoform, we performed rescue experiments in resistant cells by re-expressing AMOT-p80 following AMOT knockdown. In both SNUC5/5FUR and SNUC5/OXTR cells, siAMOT#2 (3′UTR-targeting) reduced AMOT-p80 and AMOT-p130, with lower c-Myc and Cyclin D1 protein levels. Co-transfection with an siRNA-resistant AMOT-p80 construct restored AMOT-p80, but not AMOT-p130, with concomitant recovery of c-Myc and Cyclin D1 protein levels (Figure 5A, Figures S14 and S15). Consistent with this molecular rescue, AMOT-p80 re-expression attenuated the chemosensitizing effect of AMOT knockdown. Under drug treatment conditions (5-FU, 9 μM in SNUC5/5FUR; OXA, 3 μM in SNUC5/OXTR), siAMOT#2 reduced viability relative to siControl, whereas AMOT-p80 re-expression restored viability toward control levels (Figure 5B). This rescue occurred despite persistently reduced AMOT-p130, supporting a role for AMOT-p80 in YAP-linked outputs and drug tolerance in these models.

### 3.6. AMOT-Associated Changes in c-Myc and Cyclin D1 Are Largely YAP-Dependent

**Figure 6:**
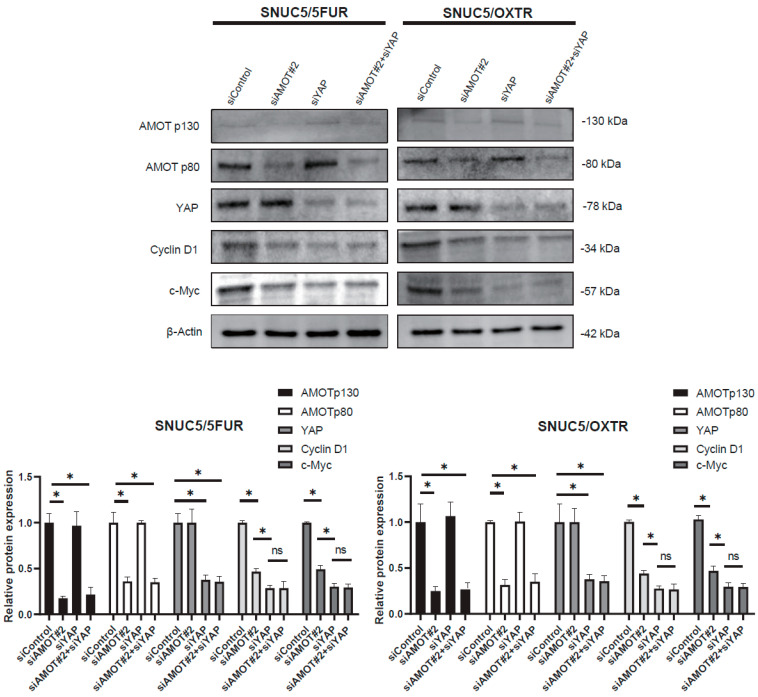
YAP knockdown reduces c-Myc/Cyclin D1 and supports a largely YAP-dependent contribution of AMOT to these outputs. SNUC5/5FUR and SNUC5/OXTR cells were transfected with siControl, siAMOT#2, siYAP, or siAMOT#2 plus siYAP. AMOT-p80, AMOT-p130, YAP, Cyclin D1, and c-Myc protein levels were assessed by immunoblotting, with semi-quantitative densitometric estimates normalized to β-actin. * *p* < 0.05. For Cyclin D1 and c-Myc, there was no significant difference (ns) between siYAP and siAMOT#2 plus siYAP.

To determine whether changes in c-Myc and Cyclin D1 following AMOT depletion involve YAP, resistant cells were transfected with siControl, siAMOT#2, siYAP, or siAMOT#2 plus siYAP. YAP knockdown reduced YAP protein levels and lowered c-Myc and Cyclin D1 protein levels. Notably, co-depletion of AMOT and YAP did not produce an additional decrease in c-Myc or Cyclin D1 beyond that observed with siYAP alone, supporting the interpretation that these AMOT-related changes are largely YAP-dependent (Figure 6, Figures S16 and S17).

### 3.7. AMOT-p80 Overexpression Increases YAP-Linked Outputs and Drug Tolerance in Parental Cells

**Figure 7:**
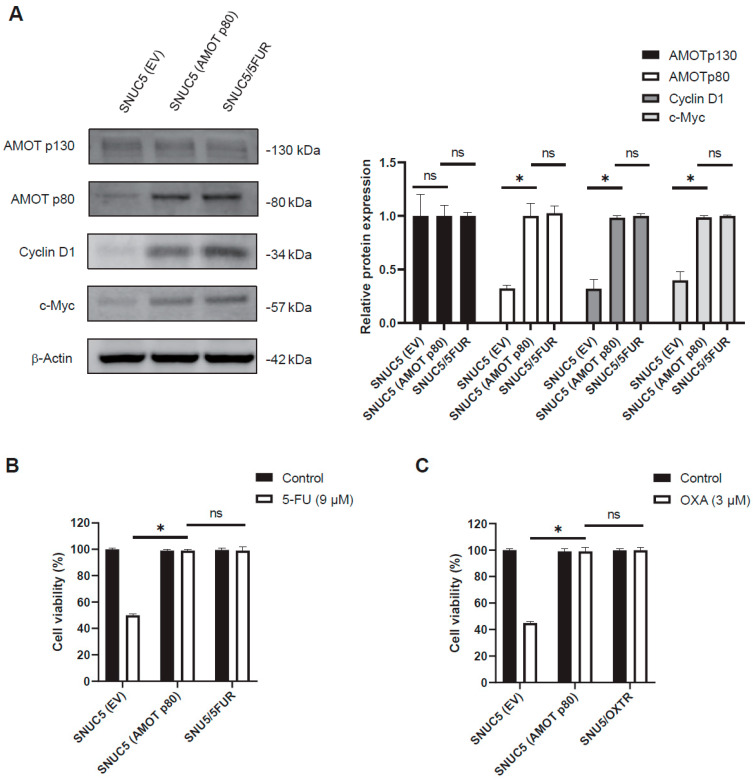
AMOT-p80 overexpression increases YAP-linked outputs, EMT-related transcription factors, and tolerance to 5-FU and oxaliplatin. (**A**) Immunoblot analysis of AMOT-p80, AMOT-p130, Cyclin D1, and c-Myc in parental SNUC5 cells transfected with empty vector (EV) or AMOT-p80, with SNUC5/5FUR cells shown for comparison; semi-quantitative densitometric estimates were normalized to β-actin. (**B**) Cell viability (MTT) of SNUC5 (EV), SNUC5 (AMOT-p80), and SNUC5/5FUR cells after 48 h treatment with 5-FU (9 μM). (**C**) Cell viability (MTT) of SNUC5 (EV), SNUC5 (AMOT-p80), and SNUC5/OXTR cells after 48 h treatment with oxaliplatin (OXA; 3 μM). * *p* < 0.05; ns, not significant.

We next tested whether AMOT-p80 overexpression in parental SNUC5 cells was linked to features of the acquired resistant state. Ectopic AMOT-p80 increased AMOT-p80 abundance and coincided with higher c-Myc and Cyclin D1 protein levels, whereas AMOT-p130 remained largely unchanged (Figure 7A and Figure S18). Functionally, AMOT-p80 overexpression increased cell viability under treatment with 5-FU (9 μM) or OXA (3 μM) relative to empty vector controls (Figure 7B,C), supporting the interpretation that AMOT-p80 expression can increase tolerance to 5-FU and OXA in parental cells.

## 4. Discussion

This study identifies AMOT-p80 as an isoform-selective contributor associated with acquired resistance to 5-fluorouracil (5-FU) and oxaliplatin (OXA) in colorectal cancer (CRC) cells. Importantly, preferential AMOT-p80 upregulation was observed not only in the OXA-resistant discovery model but also in an independently established 5-FU-resistant subline, supporting that this isoform-biased state is shared across two chemoresistant SNUC5-derived sublines rather than being restricted to a single resistant model. While AMOT family proteins are established regulators of cytoskeletal organization and Hippo–YAP signaling, our findings extend prior CRC work that largely considered total AMOT by showing preferential upregulation of AMOT-p80 across two chemoresistant SNUC5-derived sublines and linking this p80-biased state to drug tolerance, YAP-linked proliferative outputs, and EMT-related features (Figure 1, Figure 2, Figure 3 and Figure 4). Consistent with a previous report showing that AMOT overexpression enhances the malignant behavior of colon cancer cells through YAP–ERK/PI3K–AKT signaling [22], our findings refine this framework by resolving isoform-specific regulation in acquired chemoresistance. Whereas the earlier study examined AMOT overexpression without distinguishing the relative contributions of p130 and p80, our data identify preferential AMOT-p80 upregulation and support its association with nuclear YAP-linked drug tolerance.

An important aspect of our findings is that the increased AMOT output in resistant cells did not reflect a uniform induction of all isoforms. Common AMOT pre-mRNA was elevated, consistent with transcriptional activation, whereas p130-specific pre-mRNA and mature AMOT-p130 mRNA remained largely unchanged. In contrast, AMOT-p80 was markedly increased at the mRNA level and showed a corresponding increase at the protein level (Figure 1B–D). These observations support a model in which AMOT is transcriptionally activated in chemoresistant CRC cells, but the resulting transcriptional output is biased toward the p80 isoform. Rather than reflecting a simple isoform switch, this pattern suggests preferential isoform-directed regulation superimposed on increased overall AMOT transcription. Such a regulatory mode may be particularly relevant in stress-adaptive settings, where selective reinforcement of one isoform could more effectively reshape downstream survival signaling than a global increase in total AMOT alone.

Functionally, AMOT depletion exerted minimal effects on basal viability but significantly sensitized resistant cells to 5-FU or OXA, with increased apoptotic responses under drug exposure (Figure 2). Mechanistically, total YAP abundance remained comparable between parental and resistant cells, whereas resistant cells exhibited enhanced AMOT–YAP association and increased nuclear proximity (Figure 3A–C). Consistent with increased YAP-linked proliferative output, c-Myc and Cyclin D1 protein levels were higher in resistant sublines than in parental cells (Figure 3D). Importantly, AMOT depletion reduced nuclear YAP and coincided with lower c-Myc and Cyclin D1 protein levels (Figure 3E,F), consistent with YAP/TEAD-linked transcriptional outputs described across contexts [4,23]. Several observations support a functional contribution of AMOT-p80 in this setting: (i) AMOT-p80 re-expression restored c-Myc/Cyclin D1 protein levels and drug tolerance despite persistently reduced AMOT-p130 (Figure 5), (ii) AMOT-p80 overexpression in parental cells was associated with higher c-Myc/Cyclin D1 protein levels and increased drug tolerance (Figure 7), and (iii) YAP knockdown reduced c-Myc/Cyclin D1 protein levels and blunted the additional effect of AMOT depletion, consistent with epistatic behavior and supporting YAP dependence of these downstream outputs (Figure 6). Together, these data support the view that AMOT-p80 is a functionally relevant contributor to YAP-linked adaptive signaling.

AMOT proteins have long been positioned as context-dependent regulators of Hippo signaling whose effects on YAP/TAZ may vary according to isoform, localization, and cellular state [9,10]. AMOT-p130 contains an N-terminal extension harboring multiple L/PPxY motifs capable of engaging YAP WW domains, and in several systems p130 has been linked to cytoplasmic or junctional sequestration of YAP and attenuation of YAP/TAZ transcriptional outputs [10,24]. Consistent with this framework, angiomotin family proteins have also been reported to restrict canonical YAP/TAZ target gene expression in epithelial settings [10,25]. By contrast, AMOT-p80 lacks this N-terminal PPxY-rich region and has been associated with adhesion/cytoskeletal scaffolding, motility-related phenotypes, and, in some contexts, enhanced nuclear YAP activity and induction of YAP-responsive genes [9,14,17]. Our results are consistent with this latter framework. In chemoresistant CRC sublines characterized by preferential AMOT-p80 upregulation, total AMOT depletion reduced nuclear YAP and coincided with lower downstream YAP-linked outputs, including c-Myc and Cyclin D1 (Figure 3), whereas reintroduction of AMOT-p80 restored c-Myc and Cyclin D1 protein levels and drug tolerance (Figure 5). Collectively, these findings support the interpretation that a p80-biased AMOT state contributes to the maintenance of nuclear YAP-associated transcriptional programs in acquired chemoresistance [9]. At the same time, our data do not distinguish whether AMOT-p80 directly competes with AMOT-p130 for YAP association or instead acts through functionally distinct subcellular pools. Given the context-dependent roles of Motin family proteins in Hippo-YAP regulation, cytoskeletal organization, and subcellular compartmentalization, the resistant-state phenotype observed here may be better interpreted as a reorganization of AMOT–YAP-associated signaling rather than a simple displacement model [26]. Thus, the present findings are more consistent with a context-dependent shift in AMOT-associated signaling architecture in resistant cells.

Beyond proliferative YAP-linked outputs, our data also connect AMOT-p80 to EMT-related transcriptional changes and motility in resistant cells. AMOT depletion was accompanied by lower SLUG, ZEB1, and TWIST1 protein levels and impaired scratch-wound closure under proliferation-limiting conditions (Figure 4). These findings are compatible with the broader literature linking YAP activity to EMT-associated transcriptional programs and motility under therapeutic stress [20,21,27]. In this context, AMOT-p80/YAP-associated signaling may be linked not only to proliferative and anti-apoptotic outputs, but also to EMT-linked adaptive changes observed in drug-tolerant cells. Because chemoresistance is multifactorial, additional adaptations, including parallel survival signaling, DNA damage response remodeling, metabolic rewiring, and stemness-associated programs, are also likely to contribute to the full resistant phenotype in these sublines [1,2]. Nonetheless, our isoform-resolved data implicate AMOT-p80 as a convergent factor associated with drug tolerance, YAP-linked proliferative outputs, and EMT-related features in chemoresistant CRC cells. Because AMOT family proteins also contribute directly to cytoskeletal organization and actin-associated scaffolding [14], the impaired motility observed after AMOT depletion may reflect not only reduced YAP-linked EMT-associated outputs but also disruption of more direct structural functions of AMOT. Accordingly, the migration phenotype described here is likely to reflect multiple mechanisms.

An additional point of interest is that acute exposure of parental CRC cells to near-IC50 concentrations of 5-FU or OXA also increased AMOT-p80 protein abundance. This observation suggests that AMOT-p80 upregulation may not be restricted to chronically selected resistant cells, but may instead arise initially as an early adaptive response to chemotherapeutic stress, consistent with the broader role of YAP-associated signaling in survival, plasticity, and therapy adaptation across cancer contexts [28]. In this framework, AMOT-p80 induction may represent a survival-associated program that is transiently engaged during acute drug exposure and subsequently becomes stabilized or constitutively maintained during chronic drug selection. Although the present study was not designed to define the temporal evolution of this response, this interpretation provides a plausible conceptual link between the acute drug-induced increase in AMOT-p80 and its persistent elevation in resistant cells.

Because 5-FU and OXA act through nucleic acid damage and stress-associated cytotoxic mechanisms, it is also plausible that AMOT-p80/YAP-associated signaling intersects with broader damage-tolerance pathways. Both agents are known to induce complex cellular stress responses that may include nucleolar stress as well as DNA damage-associated signaling [29,30,31,32]. The present study did not directly evaluate canonical DNA damage response markers or nucleolar stress readouts, and therefore we do not conclude that AMOT-p80 defines a specific DNA damage signaling module. Rather, our results support the more cautious interpretation that AMOT-p80-associated YAP signaling may enhance survival under drug-induced stress conditions in which genotoxic and nucleolar stress responses are operative, in line with the broader role of YAP/TAZ signaling in adaptive stress tolerance and therapy resistance [28]. Under this view, AMOT-p80 upregulation would be expected to contribute primarily to adaptive survival capacity rather than to the initiating damage response itself.

The genetic background of the model system should also be considered when interpreting these findings. Cellular responses to 5-FU and OXA are strongly shaped by mutational context, including p53 status and other CRC-relevant alterations [1,2]. Thus, the AMOT-p80/YAP-associated mechanism described here should be interpreted within the signaling environment of the SNUC5-derived model used in this study [33]. SNU-C5 has been reported to harbor dysfunctional p53 with missense mutations affecting codons 218 and 248, and this background may influence stress adaptation and drug-response phenotypes [33,34]. In addition, SNUC5 has been reported to carry additional CRC-relevant alterations, including BRAF V600E and PIK3CA H1047R, and has been classified as an MSI-high cell-line model with a high mutation count [35]. While our results indicate that this adaptive axis operates in this cellular background, the extent to which it is modulated by p53 status, BRAF/PI3K pathway alterations, MSI status, or other oncogenic features remains unresolved. Thus, the AMOT-p80/YAP-associated axis described here should be viewed as a putative adaptive mechanism within the SNUC5-derived chemoresistant cell-line models used in this study. Broader validation across additional CRC models with distinct mutational profiles will therefore be important to define the generalizability and biological context-dependence of this mechanism.

Several limitations should be acknowledged. First, although the findings were reproduced across two independently established chemoresistant SNUC5-derived sublines, both models share the same parental background, and the conclusions are based on in vitro cell-line systems. Accordingly, validation in additional genetically independent colorectal cancer models, in vivo settings, and clinical CRC specimens will be important to strengthen generalizability and translational relevance. Second, although our data support YAP-linked outputs and nuclear YAP-associated signaling, direct measurements of YAP/TEAD transcriptional activity and Hippo pathway phosphorylation were not performed. Future studies incorporating TEAD-reporter assays and phosphorylation analyses will be needed to strengthen pathway-level interpretation. Third, because loading controls were assessed on parallel membranes for many Western blot analyses, the corresponding densitometric values should be interpreted as semi-quantitative estimates that support qualitative assessment of protein-expression patterns rather than as absolute quantitative measurements. Fourth, because the siRNAs used target sequences shared by AMOT isoforms, both AMOT-p80 and AMOT-p130 were reduced in loss-of-function experiments, limiting strict attribution of the phenotype to p80 depletion alone. Although rescue with AMOT-p80 supports a functional role for this isoform, additional isoform-resolved approaches will be needed to define its contribution more precisely. Fifth, although our pre-mRNA analyses are consistent with transcriptional activation coupled to p80-biased output, the regulatory mechanism underlying this bias remains undefined. Finally, while co-immunoprecipitation and PLA support AMOT–YAP association and nuclear proximity, they do not establish direct binding or define the relevant interaction interfaces, and the molecular basis linking AMOT-p80 upregulation to nuclear YAP maintenance remains to be clarified.

## 5. Conclusions

In conclusion, chemoresistant CRC cells exhibit transcriptionally activated AMOT regulation with preferential upregulation of AMOT-p80. This p80-biased AMOT state is associated with nuclear YAP abundance, YAP-linked proliferative outputs, EMT-related features, and tolerance to 5-FU and oxaliplatin, supporting a putative AMOT-p80/YAP-linked adaptive mechanism within the SNUC5-derived chemoresistant cell-line models.

## Data Availability

The original contributions presented in this study are included in the article. Further inquiries can be directed to the corresponding author.

## Supplementary Materials

The following supporting information can be downloaded at: https://www.mdpi.com/article/10.3390/biom16060767/s1, Figure S1: Original uncropped Western blot images corresponding to Figure 1D; Figure S2: Original uncropped Western blot images corresponding to Figure 1E; Figure S3: Original uncropped Western blot images corresponding to Figure 3A; Figure S4: Original uncropped Western blot images corresponding to the left panel of Figure 3B; Figure S5: Original uncropped Western blot images corresponding to the right panel of Figure 3B; Figure S6: Original uncropped Western blot images corresponding to Figure 3D; Figure S7: Original uncropped Western blot images corresponding to the left panel of Figure 3E; Figure S8: Original uncropped Western blot images corresponding to the right panel of Figure 3E; Figure S9: Original uncropped Western blot images corresponding to the left panel of Figure 3F; Figure S10: Original uncropped Western blot images corresponding to the right panel of Figure 3F; Figure S11: Original uncropped Western blot images corresponding to Figure 4A; Figure S12: Original uncropped Western blot images corresponding to the left panel of Figure 4B; Figure S13: Original uncropped Western blot images corresponding to the right panel of Figure 4B; Figure S14: Original uncropped Western blot images corresponding to the left panel of Figure 5A; Figure S15: Original uncropped Western blot images corresponding to the right panel of Figure 5A; Figure S16: Original uncropped Western blot images corresponding to the left panel of Figure 6; Figure S17: Original uncropped Western blot images corresponding to the right panel of Figure 6; Figure S18: Original uncropped Western blot images corresponding to Figure 7A.

## Abbreviations

5-FU, 5-fluorouracil; AMOT, angiomotin; CRC, colorectal cancer; DMEM, Dulbecco’s Modified Eagle Medium; DMSO, dimethyl sulfoxide; FACS, fluorescence-activated cell sorting; FBS, fetal bovine serum; HRP, horseradish peroxidase; IC_50_, half-maximal inhibitory concentration; OXA, oxaliplatin; PBS, phosphate-buffered saline; PI, propidium iodide; qRT-PCR, quantitative reverse transcription polymerase chain reaction; SD, standard deviation; siRNA, small interfering RNA; WB, Western blot.
