# Supplementary material for: Preferential Upregulation of AMOT-p80 Is Associated with YAP-Linked Resistance to 5-Fluorouracil and Oxaliplatin in Colorectal Cancer Cells
Source: Biomolecules. 2026 May 22;16(6):767. doi: 10.3390/biom16060767 (PMC13296975; doi:10.3390/biom16060767)
Supplement: Supplementary file 1 [file biomolecules-16-00767-s001.zip › biomolecules-4254047-supplementary.pdf]

Supplementary Materials

Preferential upregulation of AMOT-p80 is associated with YAP-linked resistance to 5-fluorouracil and oxaliplatin in colorectal cancer cells

Yeho Kim, Jin-Kyung Hong, Mina Yeom, Min-Ju Kim, Jae-Hyeon Woo, Joo-Ho Shin, Tae Hyung Won, Yunjong Lee and Jeong-Yun Choi *

Department of Pharmacology, Sungkyunkwan University School of Medicine, Suwon, Gyeonggi-do, Re-public of Korea

Table of Contents

Figure S1. Original uncropped Western blot images corresponding to Figure 1D.

Figure S2. Original uncropped Western blot images corresponding to Figure 1E.

Figure S3. Original uncropped Western blot images corresponding to Figure 3A.

Figure S4. Original uncropped Western blot images corresponding to the left panel of Figure 3B.

Figure S5. Original uncropped Western blot images corresponding to the right panel of Figure 3B.

Figure S6. Original uncropped Western blot images corresponding to Figure 3D.

Figure S7. Original uncropped Western blot images corresponding to the left panel of Figure 3E.

Figure S8. Original uncropped Western blot images corresponding to the right panel of Figure 3E.

Figure S9. Original uncropped Western blot images corresponding to the left panel of Figure 3F.

Figure S10. Original uncropped Western blot images corresponding to the right panel of Figure 3F.

Figure S11. Original uncropped Western blot images corresponding to Figure 4A.

Figure S12. Original uncropped Western blot images corresponding to the left panel of Figure 4B.

Figure S13. Original uncropped Western blot images corresponding to the right panel of Figure 4B.

Figure S14. Original uncropped Western blot images corresponding to the left panel of Figure 5A.

Figure S15. Original uncropped Western blot images corresponding to the right panel of Figure 5A.

Figure S16. Original uncropped Western blot images corresponding to the left panel of Figure 6.

Figure S17. Original uncropped Western blot images corresponding to the right panel of Figure 6.

Figure S18. Original uncropped Western blot images corresponding to Figure 7A.

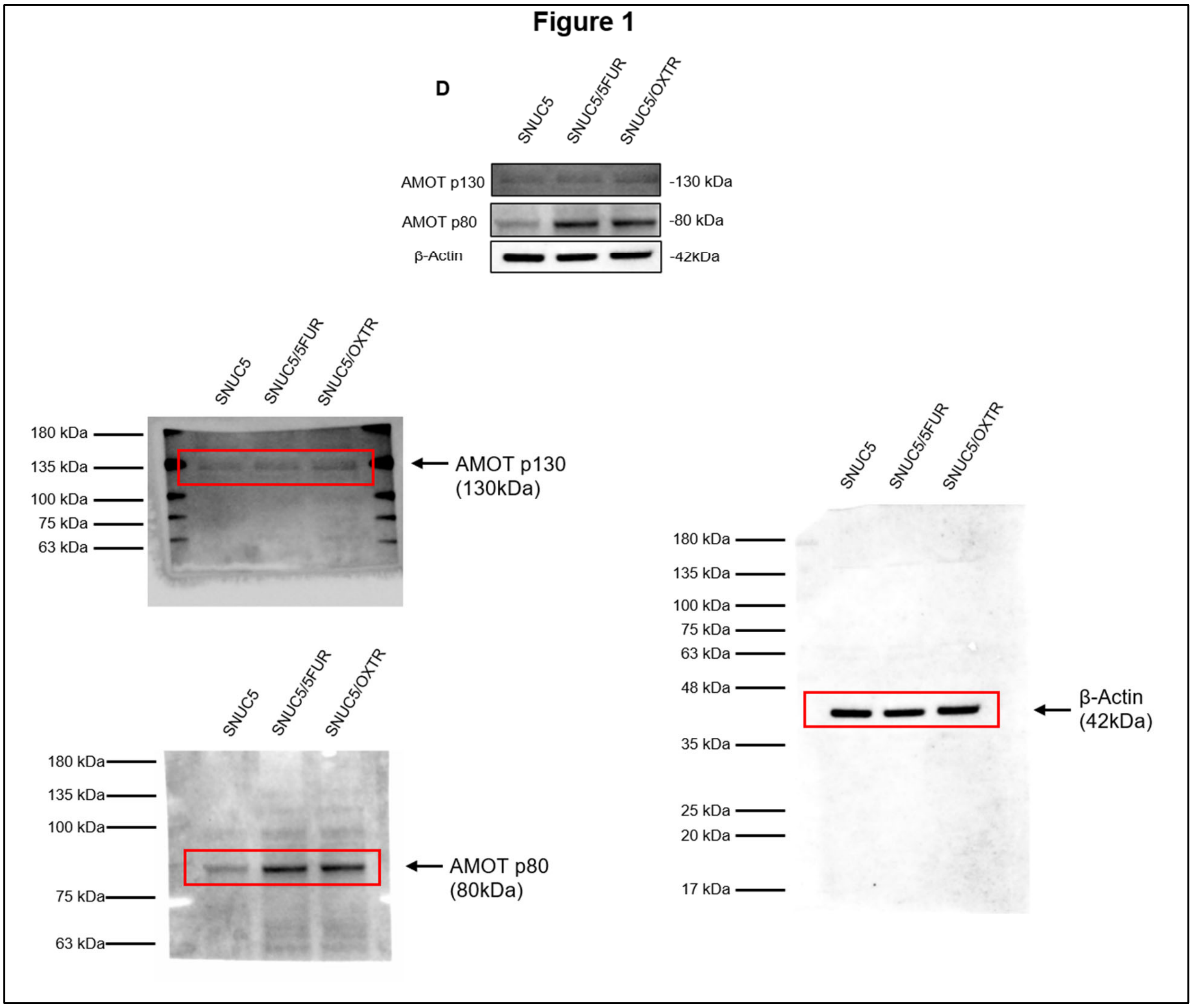

Figure S1. Original uncropped Western blot images corresponding to Figure 1D. AMOT-p80, AMOT-p130, and β-actin were assessed on separate membranes prepared from the same lysates and processed in parallel under matched conditions. Red rectangles indicate the regions shown in the assembled panel.

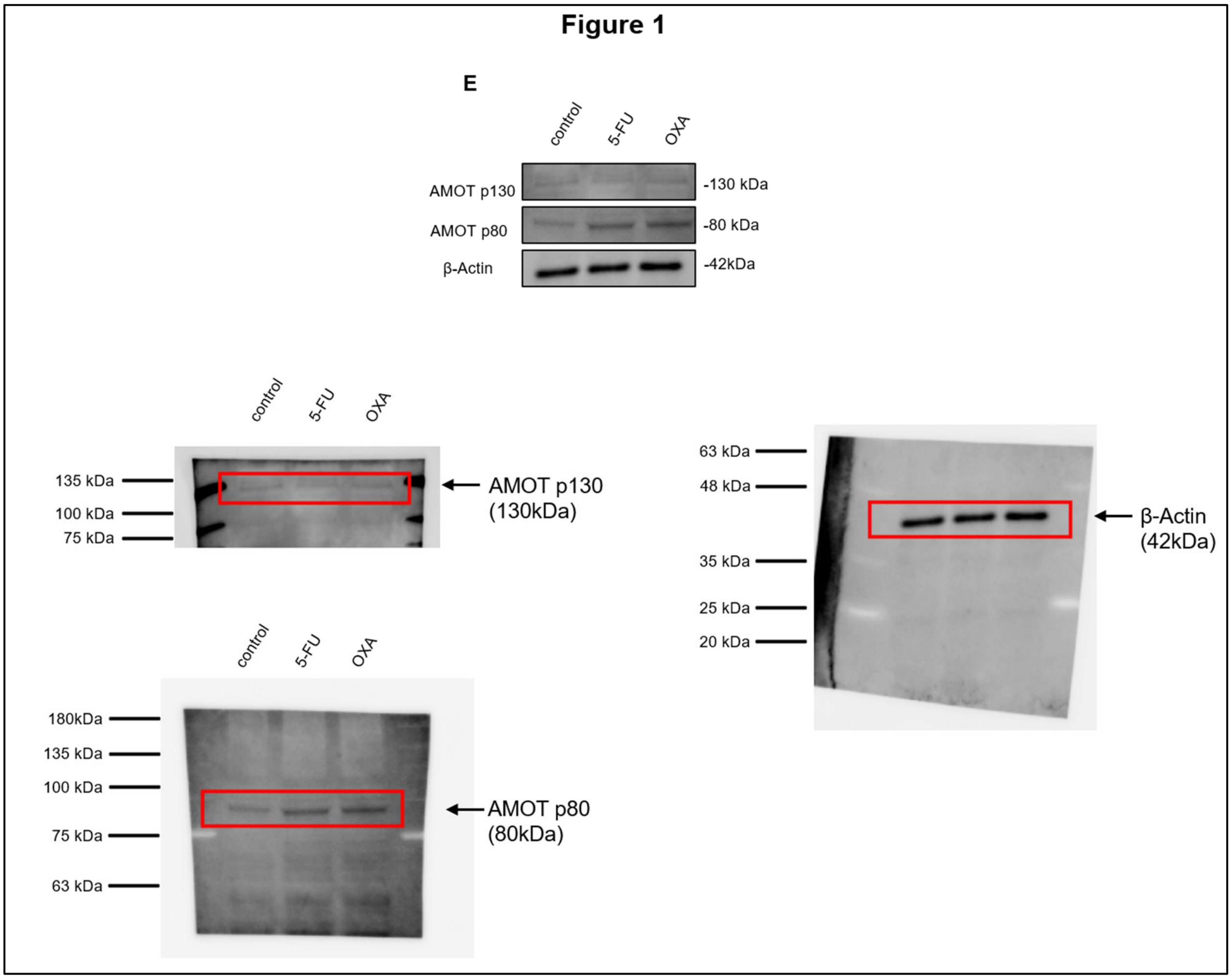

Figure S2. Original uncropped Western blot images corresponding to Figure 1E. AMOT-p80, AMOT-p130, and β-actin were assessed on separate membranes prepared from the same lysates and processed in parallel under matched conditions. Red rectangles indicate the regions shown in the assembled panel.

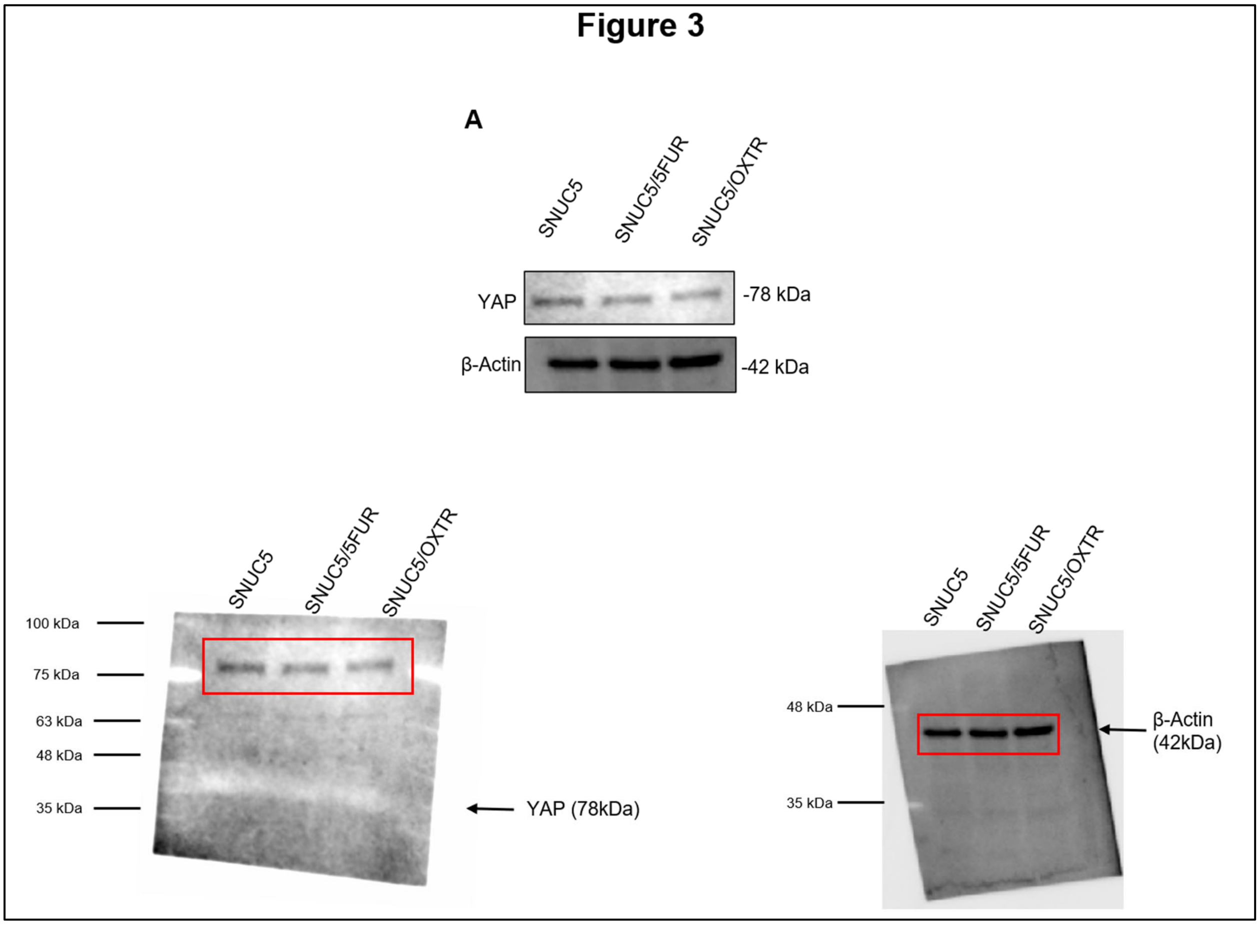

Figure S3. Original uncropped Western blot images corresponding to Figure 3A. YAP and β-actin were assessed on separate membranes prepared from the same lysates and processed in parallel under matched conditions. Red rectangles indicate the regions shown in the assembled panel.

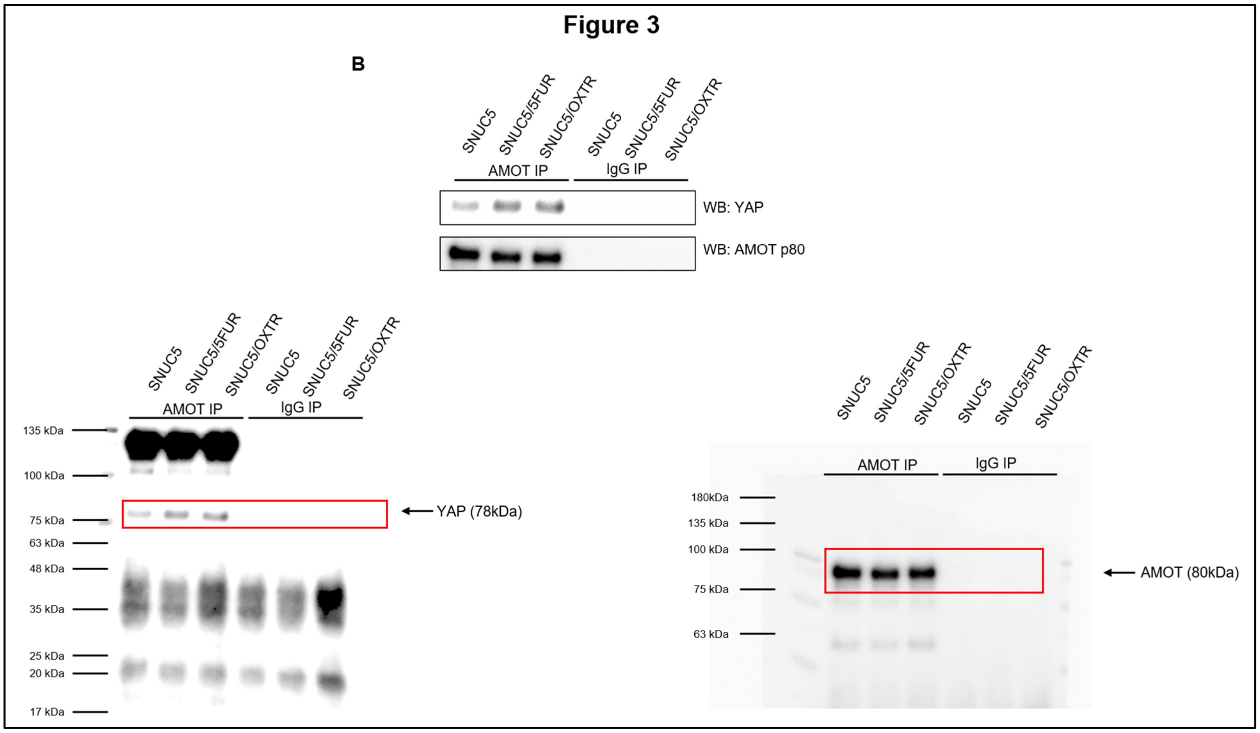

Figure S4. Original uncropped Western blot images corresponding to the left panel of Figure 3B. Co-immunoprecipitation was performed using AMOT antibody for immunoprecipitation. AMOT-p80 and YAP were detected by immunoblotting on separate membranes processed in parallel under matched conditions. Red rectangles indicate the regions shown in the assembled panel.

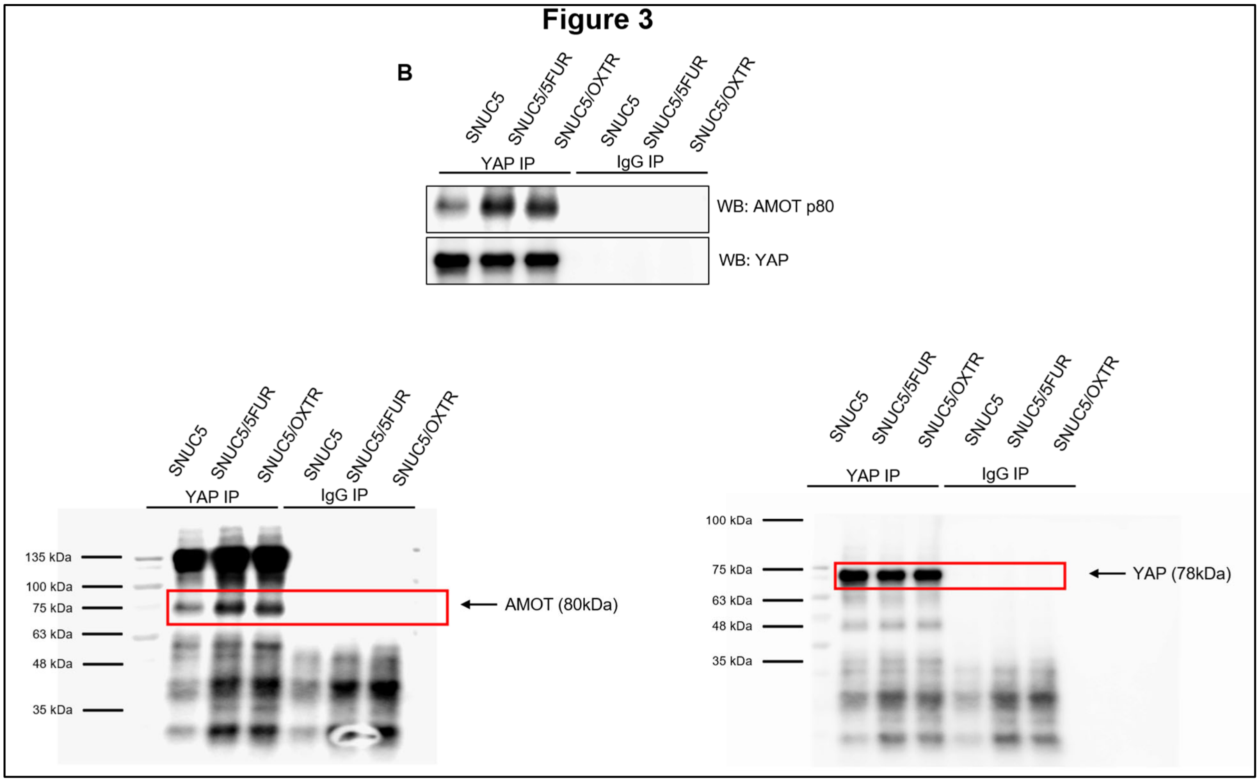

Figure S5. Original uncropped Western blot images corresponding to the right panel of Figure 3B. Co-immunoprecipitation was performed using YAP antibody for immunoprecipitation. YAP and AMOT-p80 were detected by immunoblotting on separate membranes processed in parallel under matched conditions. Red rectangles indicate the regions shown in the assembled panel.

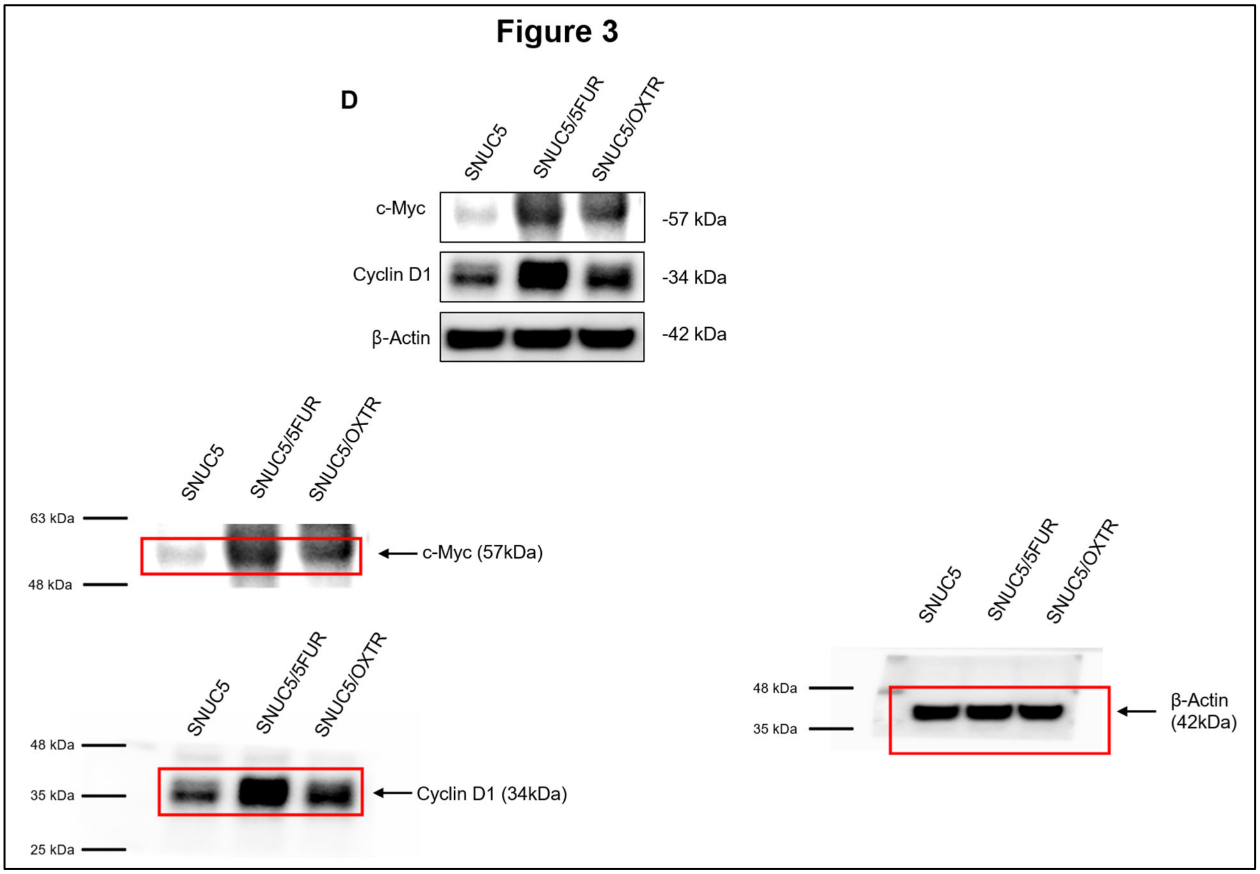

Figure S6. Original uncropped Western blot images corresponding to Figure 3D. The membrane was cut for c-Myc and cyclin D1, while β-actin was assessed on a separate membrane prepared from the same lysates and processed in parallel under matched conditions. Red rectangles indicate the regions shown in the assembled panel.

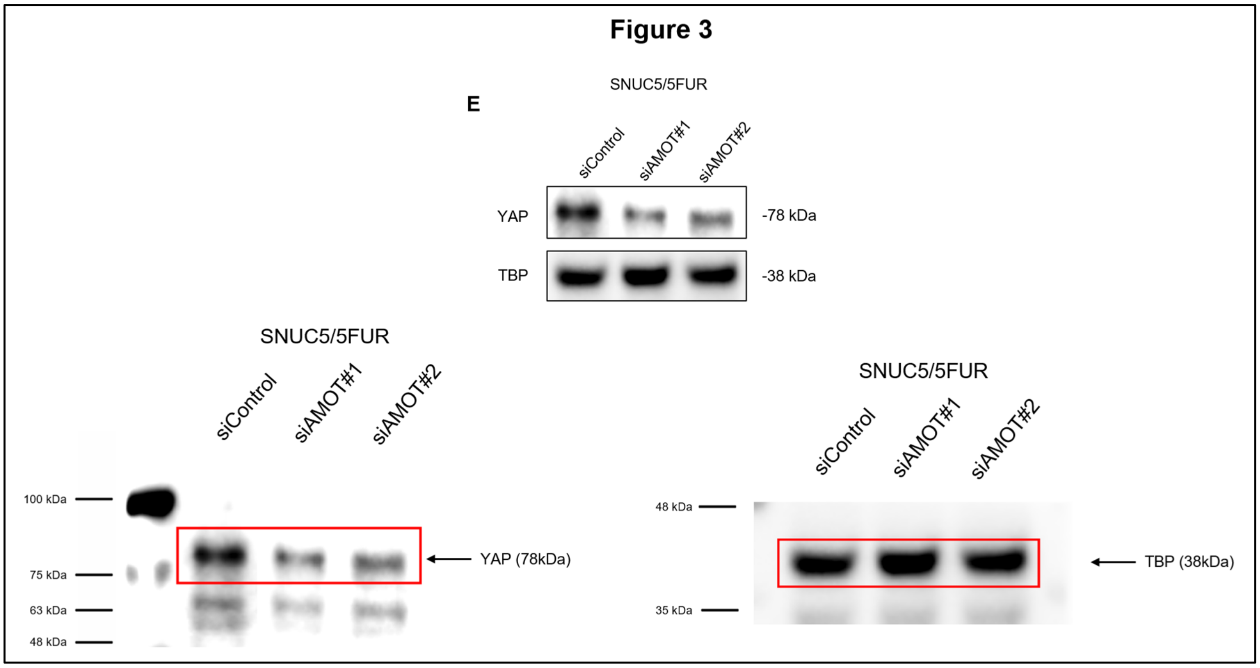

Figure S7. Original uncropped Western blot images corresponding to the left panel of Figure 3E. The membrane was cut for YAP and TBP because of their distinct molecular sizes. Red rectangles indicate the regions shown in the assembled panel.

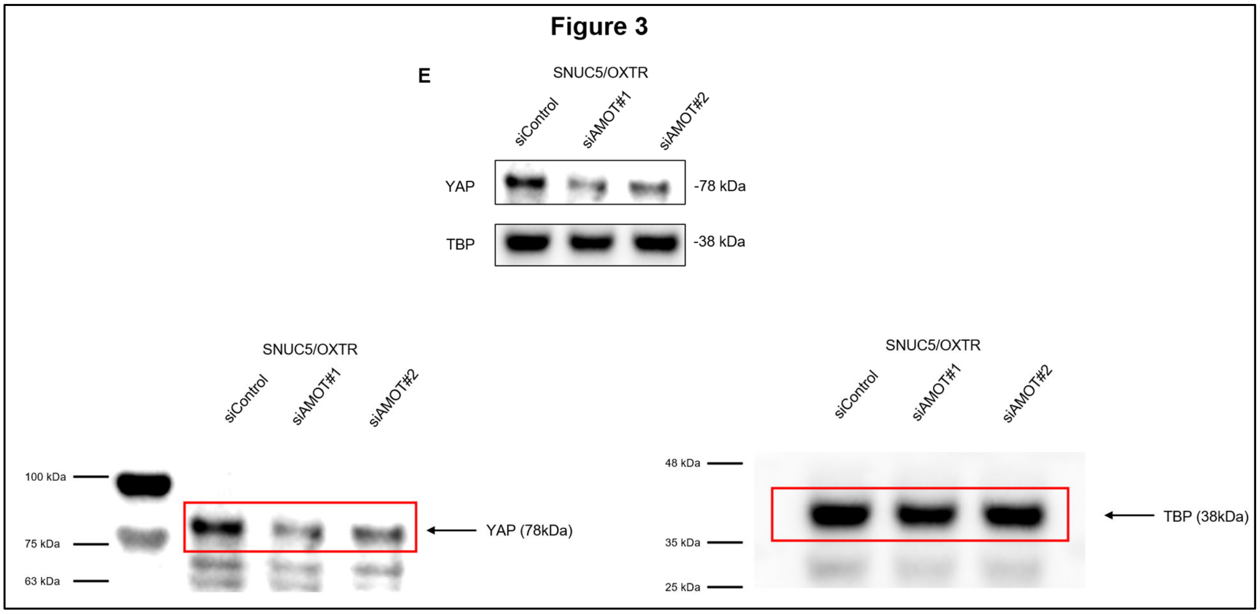

Figure S8. Original uncropped Western blot images corresponding to the right panel of Figure 3E. The membrane was cut for YAP and TBP because of their distinct molecular sizes. Red rectangles indicate the regions shown in the assembled panel.

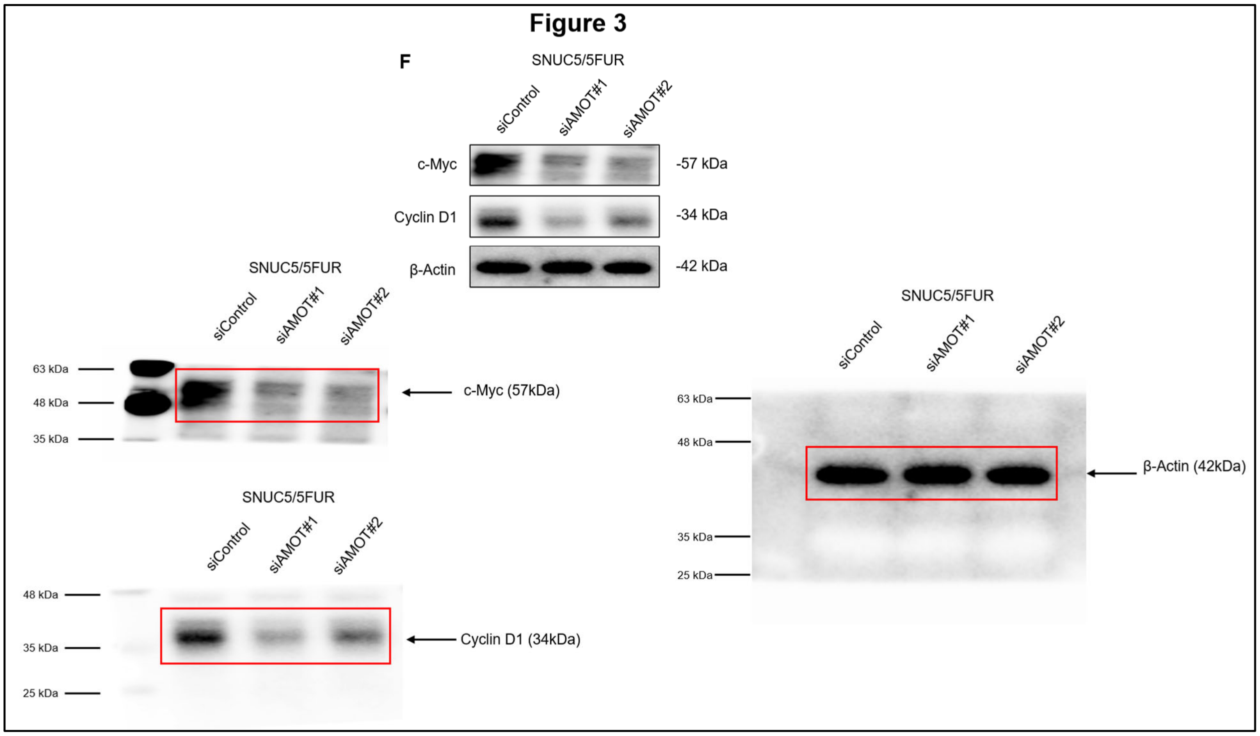

Figure S9. Original uncropped Western blot images corresponding to the left panel of Figure 3F. The membrane was cut for c-Myc and Cyclin D1, while β-actin was assessed on a separate membrane prepared from the same lysates and processed in parallel under matched conditions. Red rectangles indicate the regions shown in the assembled panel.

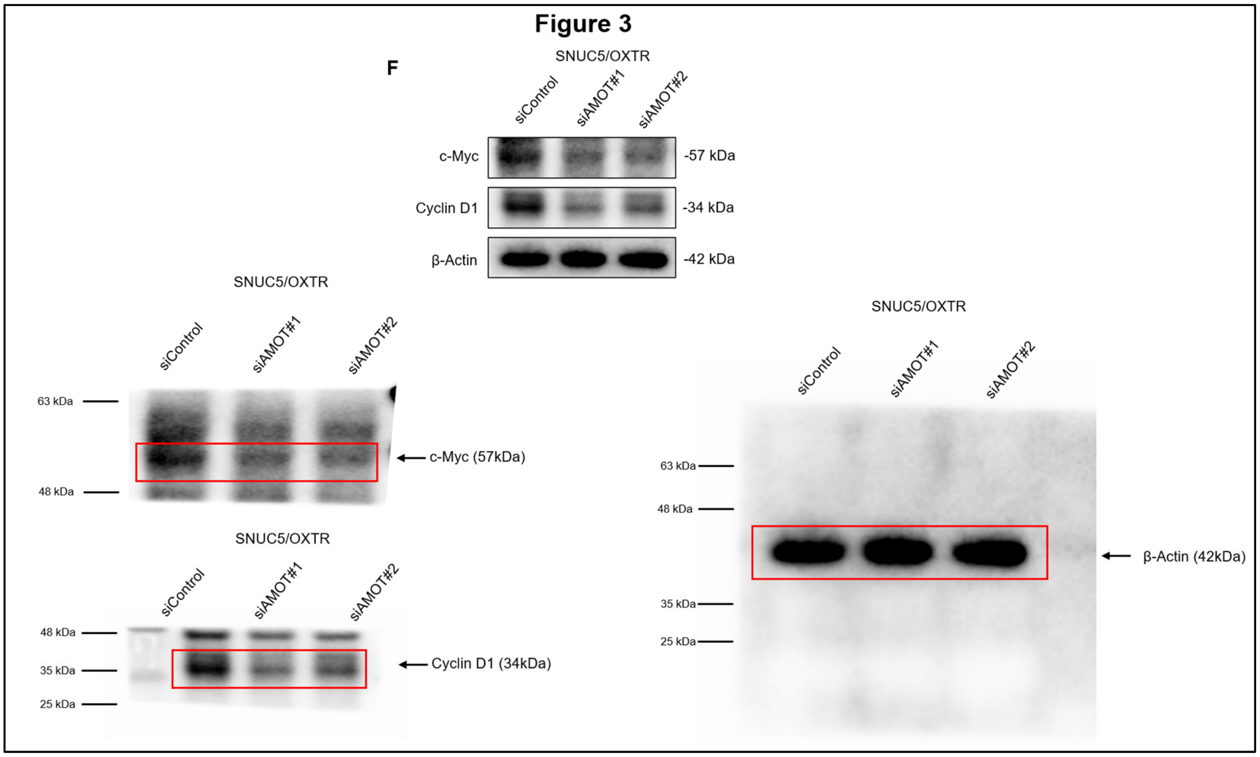

Figure S10. Original uncropped Western blot images corresponding to the right panel of Figure 3F. The membrane was cut for c-Myc and Cyclin D1, while β-actin was assessed on a separate membrane prepared from the same lysates and processed in parallel under matched conditions. Red rectangles indicate the regions shown in the assembled panel.

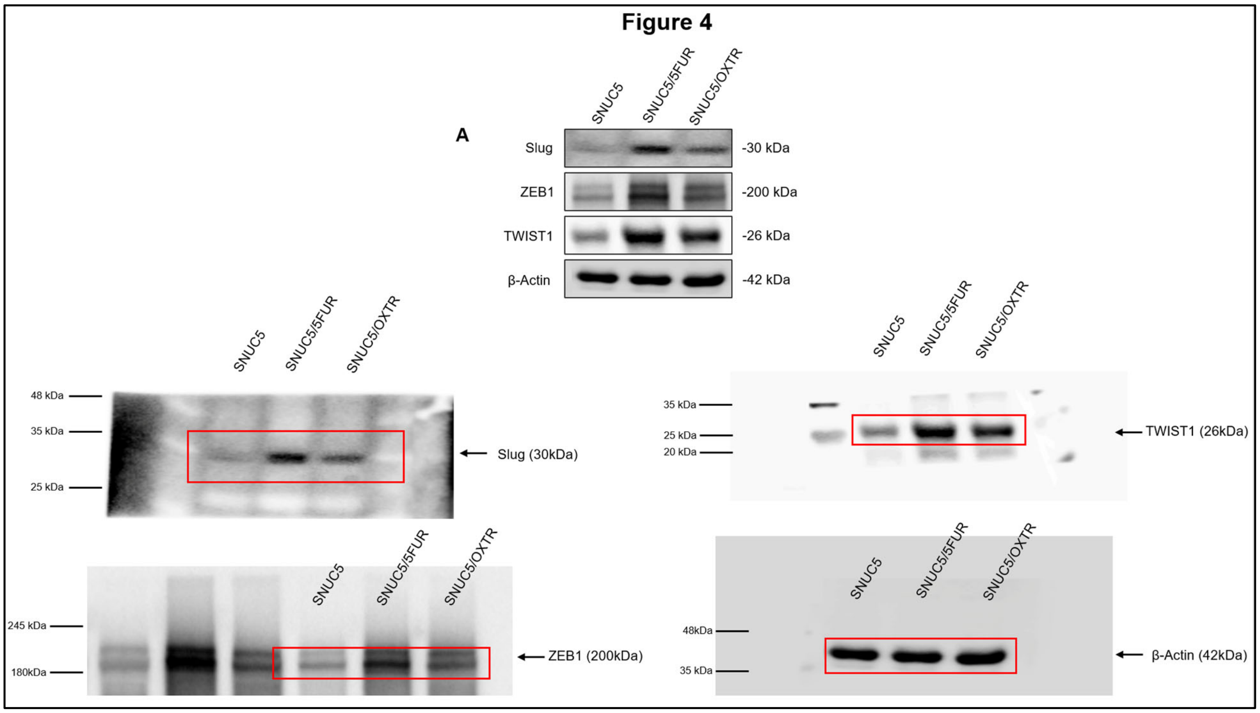

Figure S11. Original uncropped Western blot images corresponding to Figure 4A. SLUG, ZEB1, TWIST1, and β-actin were assessed on separate membranes prepared from the same lysates and processed in parallel under matched conditions. Red rectangles indicate the regions shown in the assembled panel.

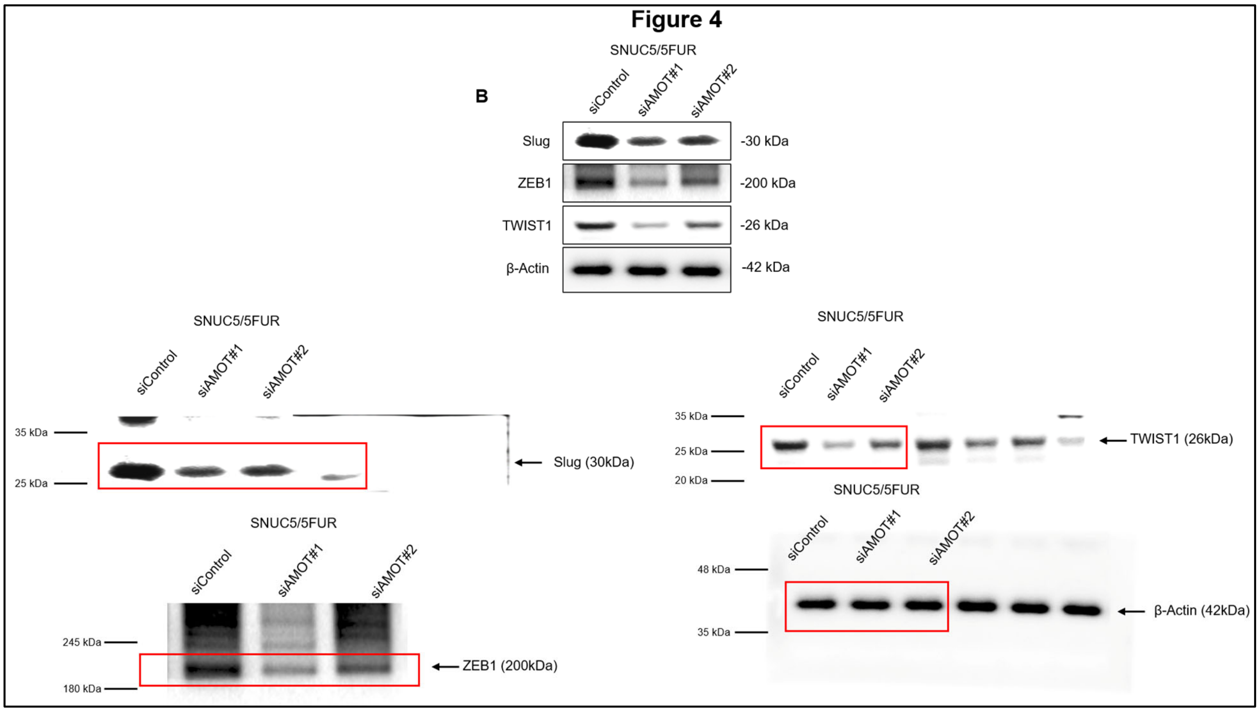

Figure S12. Original uncropped Western blot images corresponding to the left panel of Figure 4B. SLUG and ZEB1 were assessed on different sections of the same cut membrane, while TWIST1 and β-actin were assessed on different sections of a separate cut membrane; both membranes were prepared from the same lysates and processed in parallel under matched conditions. Red rectangles indicate the regions shown in the assembled panel.

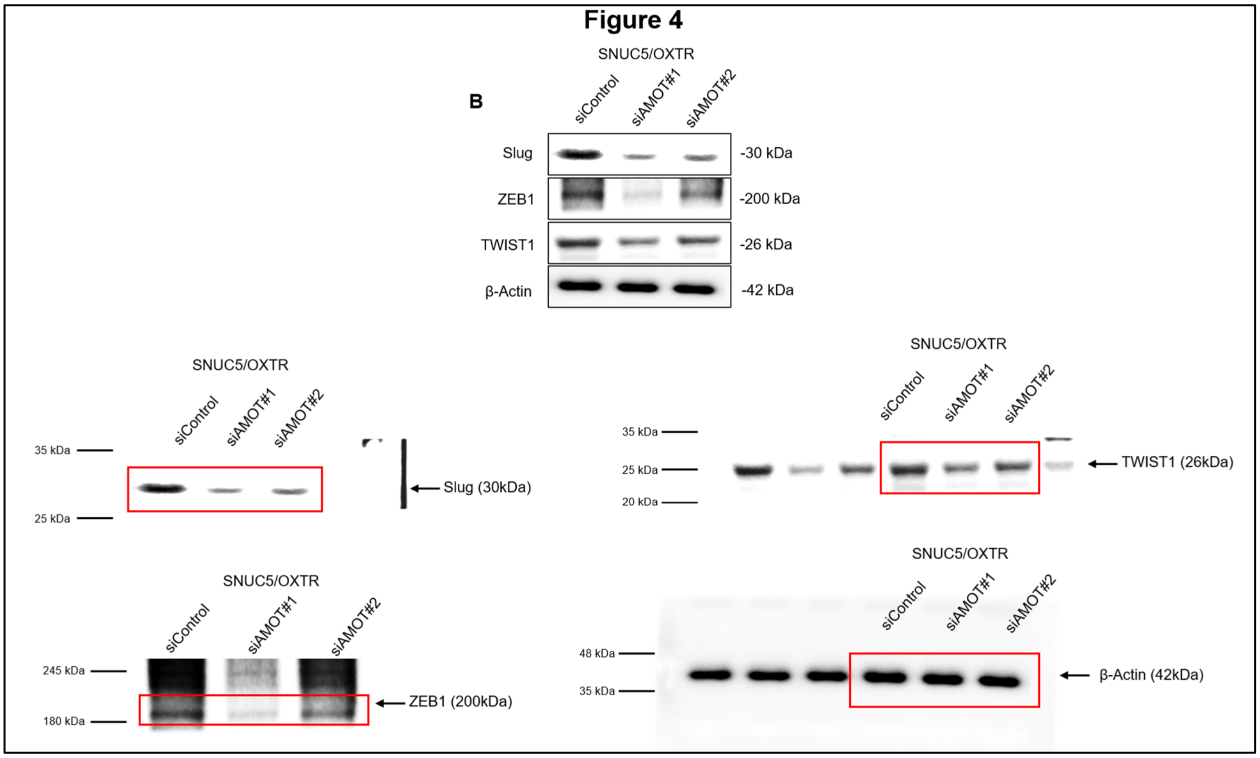

Figure S13. Original uncropped Western blot images corresponding to the right panel of Figure 4B. SLUG and ZEB1 were assessed on different sections of the same cut membrane, while TWIST1 and β-actin were assessed on different sections of a separate cut membrane; both membranes were prepared from the same lysates and processed in parallel under matched conditions. Red rectangles indicate the regions shown in the assembled panel.

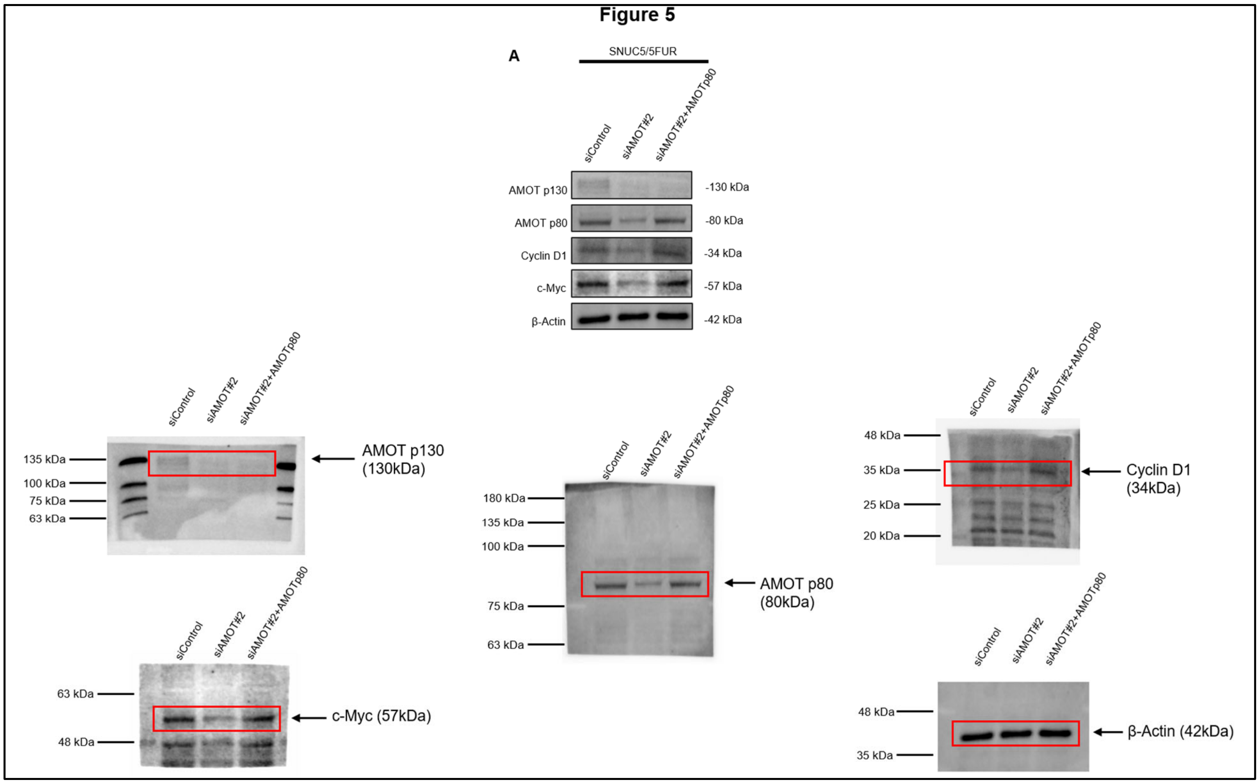

Figure S14. Original uncropped Western blot images corresponding to the left panel of Figure 5A. AMOT-p80, AMOT-p130, Cyclin D1, c-Myc, and β-actin were assessed on separate membranes prepared from the same lysates and processed in parallel under matched conditions. Red rectangles indicate the regions shown in the assembled panel.

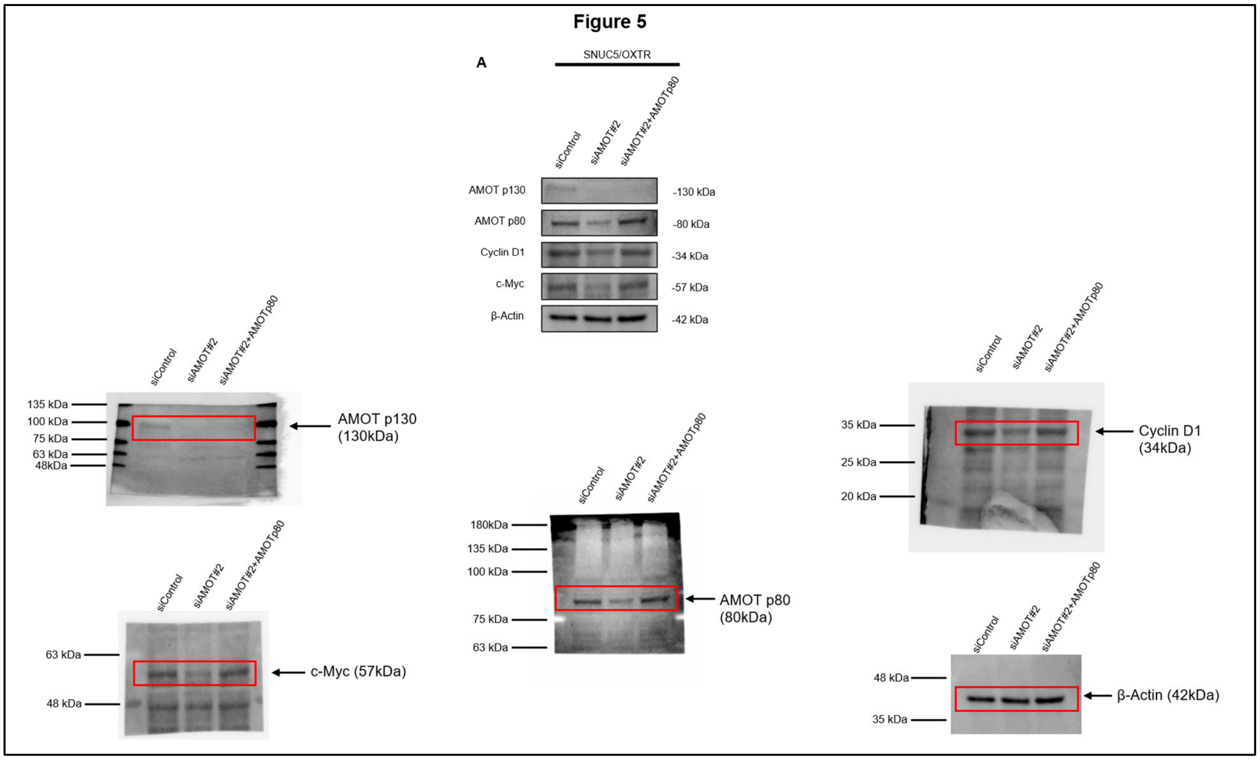

Figure S15. Original uncropped Western blot images corresponding to the right panel of Figure 5A. AMOT-p80, AMOT-p130, Cyclin D1, c-Myc, and β-actin were assessed on separate membranes prepared from the same lysates and processed in parallel under matched conditions. Red rectangles indicate the regions shown in the assembled panel.

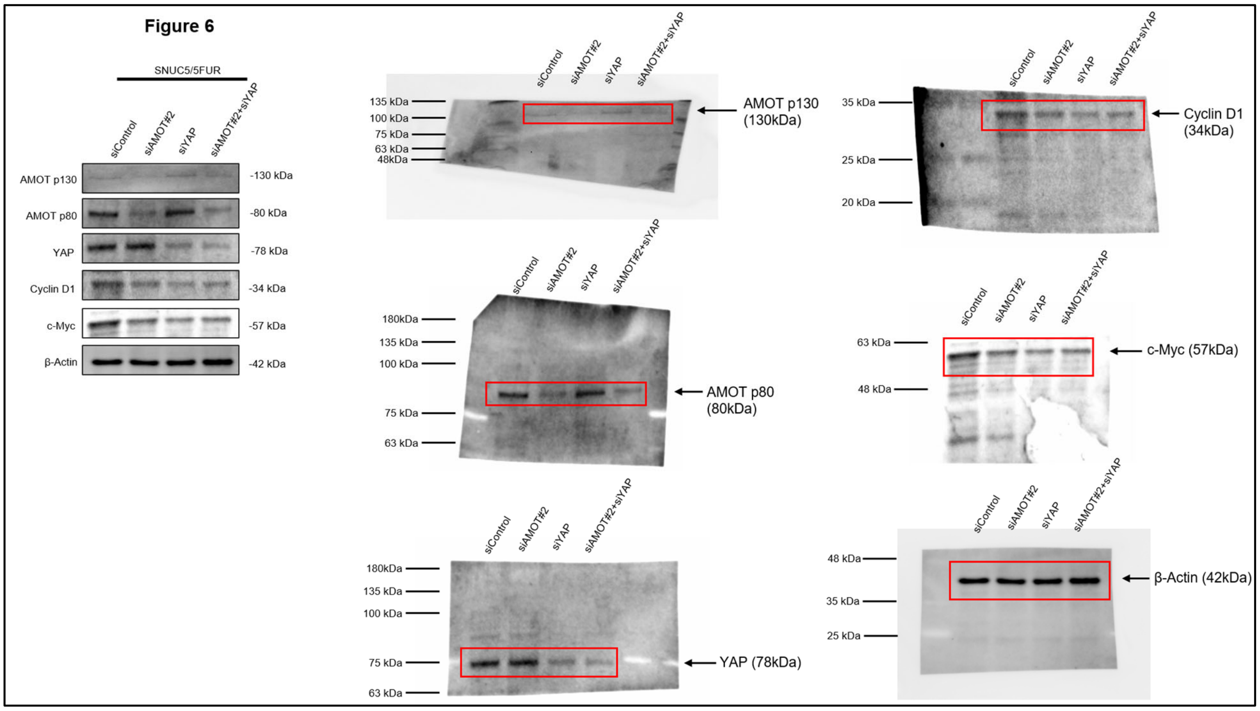

Figure S16. Original uncropped Western blot images corresponding to the left panel of Figure 6. AMOT-p80, AMOT-p130, YAP, Cyclin D1, c-Myc, and β-actin were assessed on separate membranes prepared from the same lysates and processed in parallel under matched conditions. Red rectangles indicate the regions shown in the assembled panel.

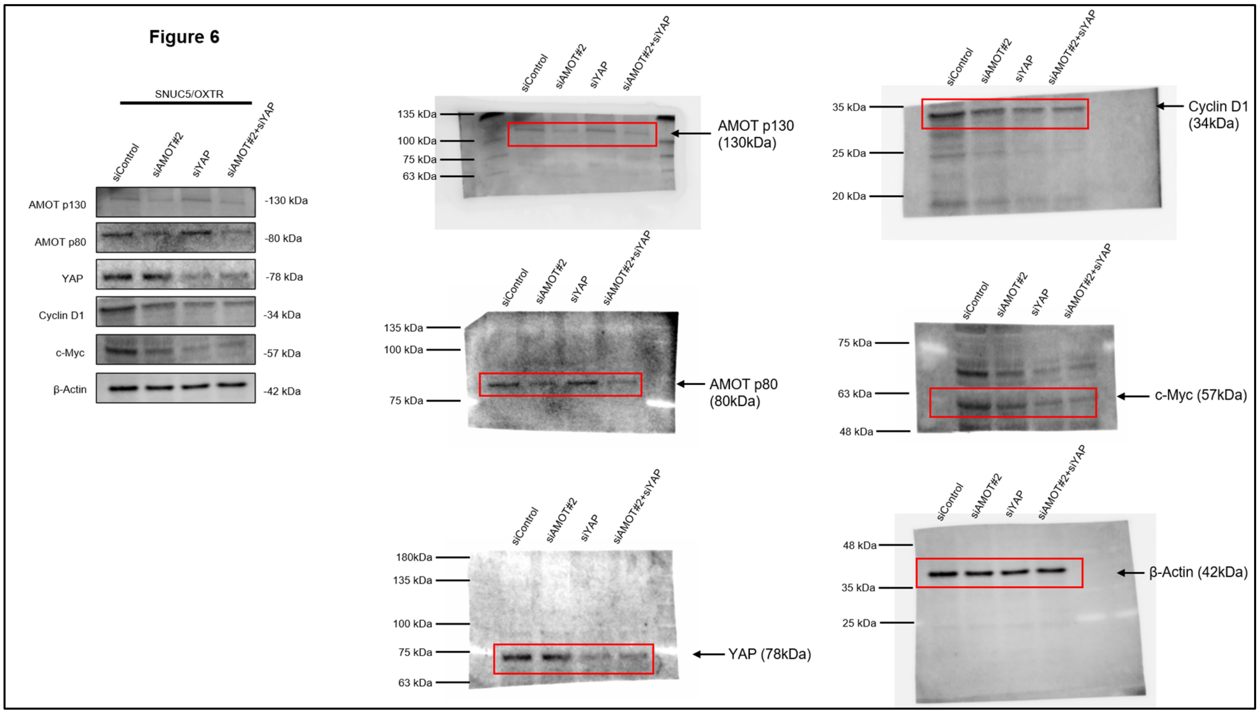

Figure S17. Original uncropped Western blot images corresponding to the right panel of Figure 6. AMOT-p80, AMOT-p130, YAP, Cyclin D1, c-Myc, and β-actin were assessed on separate membranes prepared from the same lysates and processed in parallel under matched conditions. Red rectangles indicate the regions shown in the assembled panel.

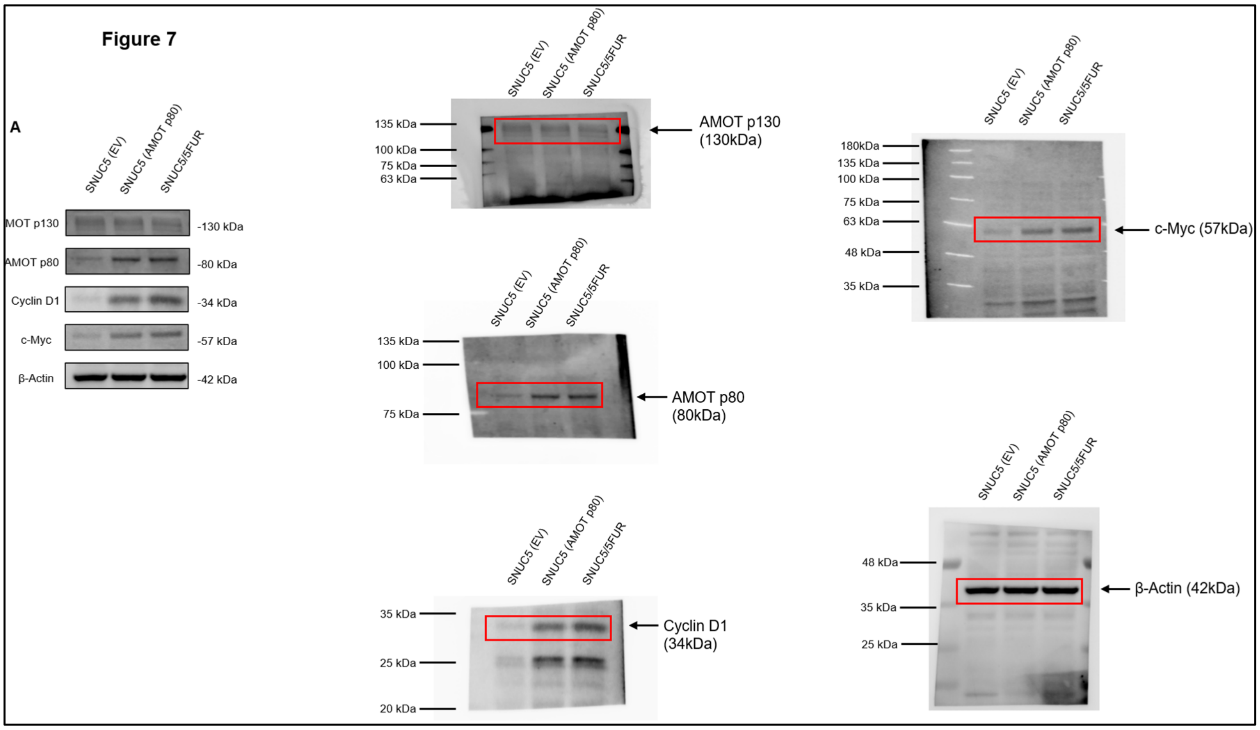

Figure S18. Original uncropped Western blot images corresponding to Figure 7A. AMOT-p80, AMOT-p130, Cyclin D1, c-Myc, and β-actin were assessed on separate membranes prepared from the same lysates and processed in parallel under matched conditions. Red rectangles indicate the regions shown in the assembled panel.
